# Patterns of Brain Maturation in Autism and Their Molecular Associations

**DOI:** 10.1001/jamapsychiatry.2024.3194

**Published:** 2024-10-16

**Authors:** Charlotte M. Pretzsch, Martina Arenella, Jason P. Lerch, Michael V. Lombardo, Christian Beckmann, Tim Schaefer, Johanna Leyhausen, Caroline Gurr, Anke Bletsch, Lisa M. Berg, Hanna Seelemeyer, Dorothea L. Floris, Bethany Oakley, Eva Loth, Thomas Bourgeron, Tony Charman, Jan Buitelaar, Grainne McAlonan, Declan Murphy, Christine Ecker

**Affiliations:** 1 Department of Forensic and Neurodevelopmental Sciences, Institute of Psychiatry, Psychology, and Neuroscience, King’s College London, London, United Kingdom; 2 Wellcome Centre for Integrative Neuroimaging, Nuffield Department of Clinical Neuroscience, University of Oxford, Oxford, United Kingdom; 3 Laboratory for Autism and Neurodevelopmental Disorders, Center for Neuroscience and Cognitive Systems, Istituto Italiano di Tecnologia, Rovereto, Italy; 4 Department of Cognitive Neuroscience, Donders Institute for Brain, Cognition, and Behaviour, Radboud University Medical Centre, Nijmegen, the Netherlands; 5 Department of Child and Adolescent Psychiatry, University Hospital Goethe University, Frankfurt am Main, Germany; 6 Department of Biosciences, Goethe University Frankfurt, Frankfurt am Main, Germany; 7 Brain Imaging Center, Goethe-University, Frankfurt am Main, Germany; 8 Methods of Plasticity Research, Department of Psychology, University of Zurich, Zurich, Switzerland; 9 Institut Pasteur, Human Genetics and Cognitive Functions Unit, Paris, France; 10 Department of Psychology, Institute of Psychiatry, Psychology and Neuroscience, King’s College London, London, United Kingdom

## Abstract

**Importance:**

In the neurotypical brain, regions develop in coordinated patterns, providing a fundamental scaffold for brain function and behavior. Whether altered patterns contribute to clinical profiles in neurodevelopmental conditions, including autism, remains unclear.

**Objectives:**

To examine if, in autism, brain regions develop differently in relation to each other and how these differences are associated with molecular/genomic mechanisms and symptomatology.

**Design, Setting, and Participants:**

This study was an analysis of one the largest deep-phenotyped, case-control, longitudinal (2 assessments separated by approximately 12-24 months) structural magnetic resonance imaging and cognitive-behavioral autism datasets (EU-AIMS Longitudinal European Autism Project [LEAP]; study dates, February 2014-November 2017) and an out-of-sample validation in the Brain Development Imaging Study (BrainMapASD) independent cohort. Analyses were performed during the 2022 to 2023 period. This multicenter study included autistic and neurotypical children, adolescents, and adults. Autistic participants were included if they had an existing autism diagnosis (*DSM-IV*/*International Statistical Classification of Diseases and Related Health Problems, Tenth Revision *or *DSM-5* criteria). Autistic participants with co-occurring psychiatric conditions (except psychosis/bipolar disorder) and those taking regular medications were included.

**Exposures:**

Neuroanatomy of neurotypical and autistic participants.

**Main Outcomes and Measures:**

Intraindividual changes in surface area and cortical thickness over time, analyzed via surface-based morphometrics.

**Results:**

A total of 386 individuals in the LEAP cohort (6-31 years at first visit; 214 autistic individuals, mean [SD] age, 17.3 [5.4] years; 154 male [72.0%] and 172 neurotypical individuals, mean [SD] age, 16.35 [5.7] years; 108 male [62.8%]) and 146 individuals in the BrainMapASD cohort (11-18 years at first visit; 49 autistic individuals, mean [SD] age, 14.31 [2.4] years; 42 male [85.7%] and 97 neurotypical individuals, mean [SD] age, 14.10 [2.5] years; 58 male [59.8%]). Maturational between-group differences in cortical thickness and surface area were established that were mostly driven by sensorimotor regions (eg, across features, absolute loadings for early visual cortex ranged from 0.07 to 0.11, whereas absolute loadings for dorsolateral prefrontal cortex ranged from 0.005 to 0.06). Neurodevelopmental differences were transcriptomically enriched for genes expressed in several cell types and during various neurodevelopmental stages, and autism candidate genes (eg, downregulated genes in autism, including those regulating synaptic transmission; enrichment odds ratio =3.7; *P *=2.6 × ^−10^). A more neurotypical, less autismlike maturational profile was associated with fewer social difficulties and more typical sensory processing (false discovery rate *P *<.05; Pearson *r* ≥0.17). Results were replicated in the independently collected BrainMapASD cohort.

**Conclusions and Relevance:**

Results of this case-control study suggest that the coordinated development of brain regions was altered in autism, involved a complex interplay of temporally sensitive molecular mechanisms, and may be associated with both lower-order (eg, sensory) and higher-order (eg, social) clinical features of autism. Thus, examining maturational patterns may provide an analytic framework to study the neurobiological origins of clinical profiles in neurodevelopmental/mental health conditions.

## Key Points

**Question:**

In autistic individuals (compared with neurotypical individuals), do brain regions develop differently in relation to each other, and are these differences associated with molecular/genomic mechanisms and symptomatology?

**Findings:**

In this longitudinal case-control study including 386 individuals in the Longitudinal European Autism Project and 146 individuals in the BrainMapASD cohort, spatial patterns of developmental between-group differences in cortical thickness and surface area were established; these differences were driven mostly by sensorimotor regions and were transcriptomically enriched for genes expressed in multiple cell types, neurodevelopmental stages, and autism candidate genes. Maturational profiles that were more neurotypical and less autismlike were associated with fewer social difficulties and more typical sensory processing.

**Meaning:**

Results suggest that maturational patterns may provide an analytic framework to study neurobiological origins of clinical profiles in neurodevelopmental and mental health conditions.

## Teaser

This case-control study investigates if, in autistic individuals compared with nonautistic individuals, brain regions develop differently in relation to each other and if these differences are associated with molecular/genomic mechanisms and symptomatology.

## Introduction

Autism is a neurodevelopmental condition estimated to occur in 1 of 44 children.^1^ It is characterized by differences in social communication, restricted and repetitive patterns of interests and behaviors, and altered sensory processing.^2^ These cognitive-behavioral features vary significantly between individuals and across the human life span. This interindividual and intraindividual heterogeneity hampers diagnosis, prediction of clinical outcomes, and the delivery of effective support. A better understanding of the neurobiological associations of clinical profiles in autism across development may help parse this heterogeneity.

Previous research has shown that the autistic brain develops atypically and that maturational differences may be linked to genomic and clinical variation.^3,4,5^ For instance, studies have reported whole-brain and regional differences in the growth trajectories of multiple morphometric features, including cortical thickness and surface area.^4^ Moreover, using imaging transcriptomics, which map neuroanatomy to gene expression in silico, we have traced divergent cortical thickness and surface area development to genetic variation in specific cell types (eg, microglia).^5^ Similarly, other studies have linked altered cortical thickness and surface area in autism to differential gene expression (eg, in neuronal progenitor cells) in vivo.^6^ Finally, we and others have linked atypical cortical thickness and surface area development in specific brain regions (eg, parietal/occipital cortex) to varying clinical profiles (eg, adaptive outcome).^5^ Collectively, these studies were important first steps in delineating the longitudinal cortical neuroanatomical (and associated molecular) associations of autism core and associated features.

However, these prior studies largely examined development of individual brain regions/spatial locations on the cortical surface. This is a limitation because evidence increasingly suggests that brain regions do not mature in isolation but rather, together, in a temporally and spatially coordinated (ie, synchronized) fashion. For example, studies in neurotypical individuals have identified an anterior-posterior axis of cortical development, whereby lower-order regions (eg, sensorimotor cortex) develop before higher-order areas (eg, prefrontal cortex).^7,8,9^ This pattern develops across the life span^10^ and is regulated through the temporally and regionally specific expression of genes (ie, developmental programs).^11^ It facilitates the sensory-fugal (inward) propagation of input from sensorimotor toward transmodal brain areas. This process permits the integration of basic sensory input, eg, sounds and motion, into increasingly complex representations, such as emotions and meaning, thus transforming sensation into cognition.^12^ Hence, maturational patterns, ie, how brain regions develop in relation to each other across age, provide a fundamental scaffold for brain function.

In autism, this scaffold may be altered. Historically, numerous theories of autism have suggested a central role for differences in how brain regions relate to each other, such as in the Weak Central Coherence theory,^13^ the Developmental Disconnection Syndrome theory,^14^ and the growth regulation hypothesis.^15^ More recently, cross-sectional studies in autism have identified coordinated patterns of neuroanatomical differences, including in cortical thickness and surface area, which were associated with altered prenatal genomic cortical patterning in autistic toddlers^6^ and variation in autism core and associated (eg, sensorimotor processing) features.

This led us to hypothesize that, in autistic (vs neurotypical) individuals, brain regions develop differently in relation to each other, and these differences correlate to variation in molecular/genomic mechanisms and behavior. However, to our knowledge, no study has tested this using large-scale longitudinal cohorts.

**Figure 1.**
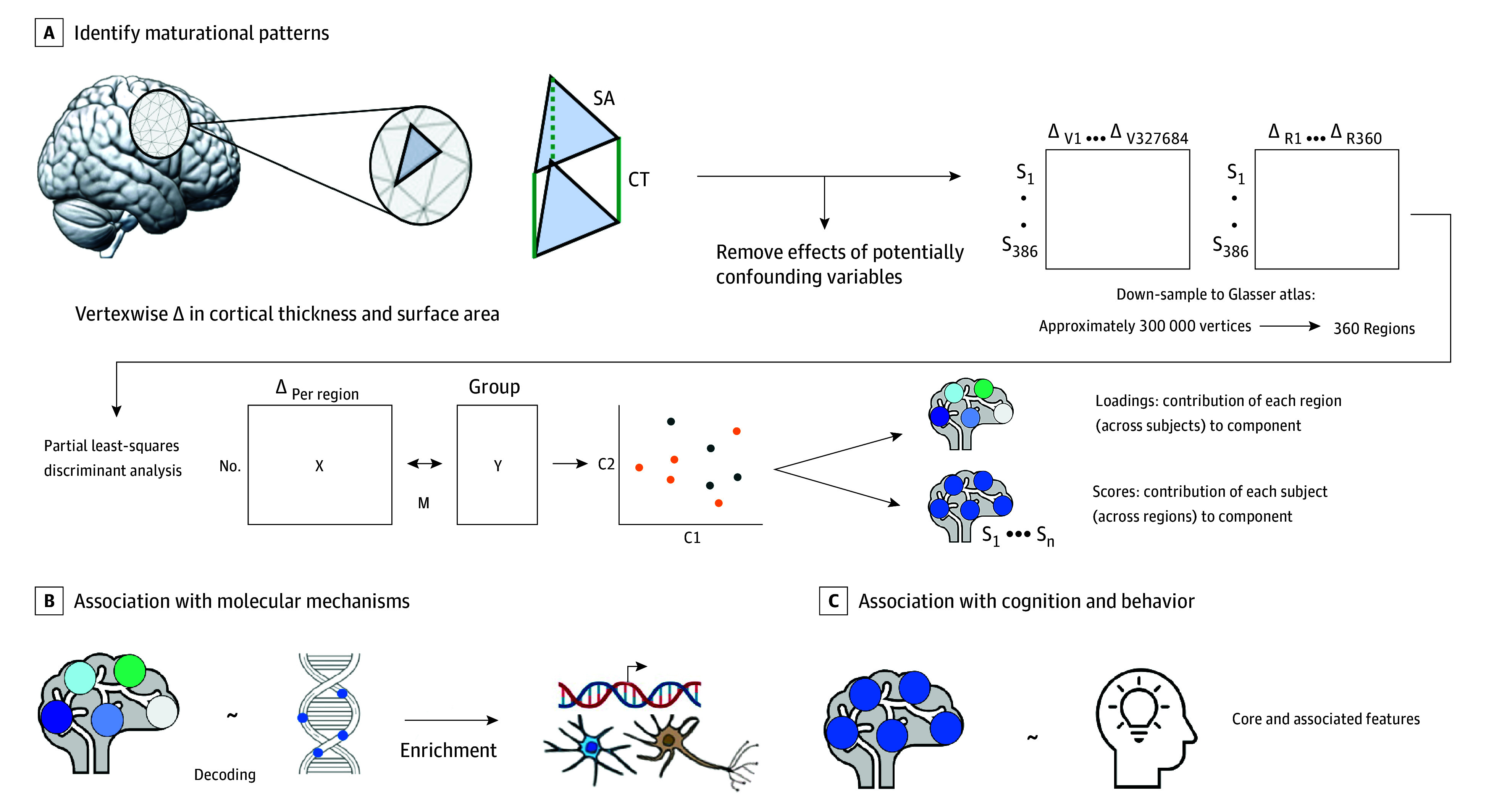
Schematic Study Overview A, Identification of differential maturational patterns, including preprocessing of neuroanatomical data (eg, removal of confounding variables through linear regression and down sampling), partial least squares-discriminant analysis to identify patterns, and extraction of loadings and scores. B, Association between differential maturational patterns (loadings) and molecular associations, including transcriptomic decoding and enrichment analyses. C, Association between differential maturational patterns (scores) and cognitive-behavioral variation. Image created with BioRender.com.

Therefore, we studied differential cortical brain development using the structural magnetic resonance imaging (MRI) and cognitive-behavioral data of individuals (both autistic and neurotypical) from one of the worldwide largest deep-phenotyped, longitudinal (2 assessments separated by approximately 12-24 months) autism datasets: the EU-AIMS Longitudinal European Autism Project (LEAP).^16^ We identified spatial patterns of developmental differences in autism using partial least squares (PLS) analyses, which map high-dimensional data (eg, morphometric measures at each point on the cortex) to lower-dimensional components that are correlated with an outcome measure (eg, diagnosis). Thus, we identified spatial patterns of differential neuroanatomical development in cortical thickness and surface area in autistic compared with neurotypical individuals. Using imaging transcriptomics,^5,17^ we explored the enrichment of these patterns for various cell types, genes linked to specific neurodevelopmental epochs/ages, and genes differentially expressed in autism. We also examined how variation in spatial patterns correlated with autism core and associated (sensory processing) features at baseline. We replicated our neuroanatomical findings in an independently collected longitudinal cohort of autistic and neurotypical individuals (Figure 1).

## Methods

### Participants

We included participants from the LEAP^3^ cohort and the independently collected longitudinal BrainMapASD project. In the original LEAP study, race and ethnicity were parent- or self-reported as Asian, Black, multiracial, White, other (information not available), or was not provided or missing. Information concerning race and ethnicity for participants in the BrainMapASD project was unavailable. Studies were approved by national/local ethics review boards at all sites and carried out to Good Clinical Practice standards. Written informed consent was obtained for all participants. Information regarding inclusion/exclusion criteria, assessments, medication status, and ethical approvals are available in the eMethods in Supplement 1. This study followed the Strengthening the Reporting of Observational Studies in Epidemiology (STROBE) reporting guidelines.

### Neuroimaging

#### MRI Data Acquisition

Using standard 3-T MRI scanners, we acquired high-resolution T1-weighted volumetric structural images with full-head coverage (field of view = 27 cm, slice thickness = 1.2 mm, in-plane resolution = 1.1 × 1.1 mm^2^) at 2 visits (mean [SD] intervisit interval: 1.6 [0.3] years) (Table 1).

**Table 1.** Image Acquisition Parameters at Each Study Site

Site	Manufacturer	Model	Software version	Acquisition sequence	Slices	TR, s	TE, ms	FA,˚	Coverage	Thickness, mm	Resolution, mm^3^	FOV, mm
Cambridge	Siemens	Verio	Syngo MR B17	Tfl3d1_ns	176	2.3	2.95	9	256 × 256^a^^,^^b^	1.2^b^	1.1 × 1.1 × 1.2^a^^,^^b^	270^b^
KCL	GE Medical Systems	Discovery mr750	LX MR DV23.1_V02_1317.c	SAG ADNI GO ACC SPGR	196	7.31	3.02	11				
Mannheim	Siemens	TimTrio	Syngo MR B17	MPRAGE ADNI	176	2.3	2.93	9				
Nijmegen	Siemens	Skyra	Syngo MR D13	Tfl3d1_16ns	176	2.3	2.93	9				
Rome	GE Medical Systems	Signa HDxt	24/LX/MR HD16.0_V02_1131.a	SAG ADNI GO ACC SPGR	172	5.96	1.76	11				
Utrecht	Philips Medical Systems	Achieva/Ingenia CX	3.2.3/3.2.3.1/ 5.1.9/5.1.9.1	ADNI GO 2	170	6.76	3.1	9				

Abbreviations: FA, flip angle; FOV, field of view; TE, echo time; TR, repetition time.

^a^ All scanners operated at 3 T.

^b^ Values for coverage, slice thickness, image resolution, and FOV were consistent across scanning sites.

### Cortical Reconstruction

Cortical reconstruction was performed using FreeSurfer software, version 6.0 (Martinos Center for Biomedical Imaging). Images were processed using well-validated, automated procedures depicted in the eMethods in Supplement 1. We computed vertexwise measures of neuroanatomical change in cortical thickness and surface area. We focused on the symmetrized percentage change, which captures the vertexwise rate of change in feature *X* with respect to the average of feature *X* across time points (eTables 2 and 7-9 in Supplement 1).

### Statistical Analysis

#### Neuroanatomical Analyses

We regressed the effects of potential confounders (age at time point 1 [T1] and its interaction with the follow-up duration, IQ, sex, and total brain measures) from our data and down-sampled the resulting residuals from 327 684 vertices to 360 regions (Glasser atlas^18^) to make our results more interpretable (in the context of existing work, including the Human Connectome Project^19^) and computationally feasible. We selected the Glasser parcellation because it is well established, validated, based on multiple imaging modalities (unlike other commonly used unimodal atlases^20,21,22^), and it balances spatial resolution with interpretability. We provided an overview over maturation per cortical feature/hemisphere within Glasser areas (both raw data points and residuals, ie, before and after removal of confounders) in eFigures 15 to 22 in Supplement 1 and attached regionwise residuals in eTables 10 and 11 in Supplement 2. To identify patterns of maturational differences between autistic and neurotypical participants, we performed PLS analyses with a categorical dependent variable, referred to as *PLS discriminant analysis* (PLS-DA). Briefly, PLS-DA extracts a set of latent factors (components) that explain the maximum amount of covariance between the independent variable (ie, neuroanatomical development in the parcellated brain regions) and the dependent variable (here: diagnostic group, with the reference group = neurotypicals) (eTables 12 and 13 in Supplement 2).

#### Interpretation of Components

Broadly, the resulting components represent spatial patterns of developmental differences between groups, ie, those regions that, together, develop most differently in autistic vs neurotypical participants. Scores, ranging from negative to positive, indicate where an individual falls on the maturational gradient from most autismlike/least neurotypical to least autismlike/most neurotypical. Average scores of zero for the autism group suggest that the model is centered around this group, ie, that the latent components are constructed such that the average score for the autism group is zero. The deviations from zero in the neurotypical group reflect how much they differ from the autistic group. In regions with negative loadings, neurotypical individuals display a more negative neuroanatomical change (ie, either a larger decrease or smaller increase in a feature) compared with the autistic individuals. In regions with positive loadings, neurotypical individuals show a more positive change (ie, a smaller decrease or larger increase in a feature) compared with the autistic group^23^ (eMethods in Supplement 1).

We tested the replicability of our results in an independently collected longitudinal cohort (BrainMapASD) and their robustness in view of age, brain parcellation scheme, medication, and clinical profile measures (eMethods in Supplement 1).

#### Genomic Analyses

To examine the molecular associations of the identified neuroanatomical patterns, we identified genes whose spatial expression significantly correlated with these patterns (decoding) and examined the functions/roles of these genes (ie, enrichment, previously described^5,17,24,25^). We explored enrichment for various cell types, neurodevelopmental epochs/ages, and genes differentially expressed in autism. We conducted separate enrichment analyses for those genes whose expression patterns correlated positively/negatively with the neuroanatomical components for each feature, and we corrected our results for multiple comparisons (within features/gene sets) using false discovery rate (FDR) correction (FDR *P* <.05) (eMethods in Supplement 1). To further explore the roles and functions of the identified genes, we examined their enrichment for biological processes (eMethods in Supplement 1).

#### Cognitive Behavioral Analyses

We examined how the identified neuroanatomical patterns related to the cognitive behavioral profiles of the participants at baseline. We correlated participants’ scores (their alignment with each neuroanatomical pattern) with 2 sets of clinical measures: (1) the Social Responsiveness Scale 2 (SRS-2),^26^ Repetitive Behaviors Scale–Revised (RBS-R),^27^ and Short Sensory Profile (SSP)^28^ (measuring autism traits across groups) and (2) the Autism Diagnostic Interview–Revised (ADI-R)^29^ and Autism Diagnostic Observation Schedule (ADOS)^30,31^ (criterion-standard measures of autism symptom severity across core domains^32^ within the autism group only). Results were corrected for multiple comparisons across measures (FDR *P* <.05) (eMethods in Supplement 1). As our neuroanatomical results were strongly driven by sensorimotor regions, we explored the association between neuroanatomy and different aspects of sensory processing using the SSP^28^ subdomains. Statistical significance was set at a 2-sided *P* value <.05.

## Results

### Demographics

Our final sample included a total of 386 individuals in the LEAP cohort (6-31 years at first visit; 214 autistic individuals, mean [SD] age, 17.3 [5.4] years; 60 female [28.0%]; 154 male [72.0%] and 172 neurotypical individuals, mean [SD] age, 16.35 [5.7] years; 64 female [37.2%]; 108 male [62.8%]) and 146 individuals in the BrainMapASD cohort (11-18 years at first visit; 49 autistic individuals, mean [SD] age, 14.31 [2.4] years; 7 female [14.3%]; 42 male [85.7%] and 97 neurotypical individuals, mean [SD] age, 14.10 [2.5] years; 39 female [40.2%]; 58 male [59.8%]) (Table 2 and eTables 1 and 7-9 in Supplement 1). The original LEAP study also gathered parent- or self-reported measures of ethnicity. Of the 386 individuals, 7 (2%) identified as Asian, 3 (0.8%) as Black, 22 (6%) as multiracial, 291 (75%) as White, 7 (2%) as other, 2 (0.5%) declined to answer, 45 (12%) did not provide information, and data were missing for 9 (2%) participants. However, because it is unclear if or how ethnicity affects or relates to brain structure, behavior, and molecular mechanisms, in line with previous research, we did not correct our analyses for ethnicity. Diagnostic groups did not differ significantly in age at T1 or time point 2 [T2], follow-up duration, sex, mean cortical thickness, or total surface area; however, as expected, there was a significant difference in full-scale IQ (FSIQ; autism group, mean [SD] FSIQ, 101.25 [19.3]; neurotypical group, mean [SD] FSIQ, 107.05 [16.5]; *P* =.002) (Table 2). Information regarding medication is available in eTable 1 in Supplement 1.

**Table 2.** Demographics of the Longitudinal European Autism Project (LEAP) AIMS Sample

Measure at T1 unless otherwise specified	Autism (n = 214)		Neurotypical (n = 172)		Test statistic (autism vs neurotypical)	*P* value^a^
	No./total No.	Mean (SD)	No./total No.	Mean (SD)		
LEAP AIMS						
ADI comm	210/214	13.00 (5.6)	NA	NA	NA	NA
ADI RRB	210/214	4.11 (2.6)	NA	NA	NA	NA
ADI social	210/214	16.73 (6.7)	NA	NA	NA	NA
Age T1, y	214/214	17.33 (5.4)	NA	16.35 (5.7)	*F*_1_ = 2.973	.09
Age T2, y	214/214	18.93 (5.5)	NA	17.93 (5.8)	*F*_1_ = 3.053	.08
CSS RRB	190/214	4.58 (2.7)	NA	NA	NA	NA
CSS SA	190/214	5.88 (2.6)	NA	NA	NA	NA
CSS total	190/214	5.18 (2.7)	NA	NA	NA	NA
FSIQ	25 ID	101.25 (19.3)	11 ID	107.05 (16.5)	*F*_1_ = 9.804	.002
Mean CT, mm	NA	2.68 (0.1)	NA	2.69 (0.1)	*F*_1_ = 0.070	.79
RBS total (overall score)	178/214	15.61 (14.4)	98/172	1.45 (3.0)	*F*_1_ = 92.238	<.001
Sex	60 F, 154 M		64 F, 108 M		χ^2^_1_ = 3.679	.06
SRS total raw score	184/214	91.34 (31.4)	100/172	23.00 (21.2)	*F*_1_ = 378.167	<.001
SSP auditory filtering	173/214	17.47 (5.4)	93/172	25.63 (3.9)	*F*_1_ = 164.771	<.001
SSP hypersensitivity	138/214	53.40 (11.9)	89/172	67.09 (4.1)	*F*_1_ = 109.911	<.001
SSP hyposensitivity	141/214	30.98 (7.2)	88/172	37.24 (3.4)	*F*_1_ = 58.345	<.001
SSP low energy	161/214	23.84 (6.8)	94/172	28.95 (2.8)	*F*_1_ = 47.691	<.001
SSP move	150/214	12.34 (3.4)	93/172	14.30 (1.5)	*F*_1_ = 29.236	<.001
SSP tactile	151/214	26.82 (6.4)	92/172	33.54 (2.3)	*F*_1_ = 95.068	<.001
SSP taste	147/214	15.27 (4.8)	89/172	18.97 (2.1)	*F*_1_ = 47.768	<.001
SSP total (overall score)	124/214	141.48 (27.9)	83/172	179.30 (10.8)	*F*_1_ = 138.661	<.001
SSP under-responsivity	163/214	27.20 (6.4)	91/172	32.75 (3.0)	*F*_1_ = 60.010	<.001
SSP visual and auditory sensitivity	169/214	18.89 (4.9)	94/172	23.65 (2.5)	*F*_1_ = 78.893	<.001
T2-T1, y	NA	1.61 (0.3)	NA	1.59 (0.3)	*F*_1_ = 0.555	.46
Total SA, cm^2^	NA	2299.58 (238.0)	NA	2316.47 (225.0)	*F*_1_ = 0.504	.48

Abbreviations: ADI, Autism Diagnostic Interview; comm, communication subscale; CSS, Autism Diagnostic Observation Schedule calibrated severity score; CT, cortical thickness; F, female; FSIQ, full-scale IQ; ID, intellectual disability; M, male; NA, not applicable; RBS, Repetitive Behaviors Scale; RRB, Restricted and Repetitive Behavior Subscale; SA, surface area; soc, social subscale; SRS, Social Responsiveness Scale; SSP, Short Sensory Profile; T1, measure at time point 1; T2, measure at time point 2.

^a^ *P* values are not corrected for multiple comparisons.

### Neuroanatomical Results

#### Primary Results

**Figure 2.**
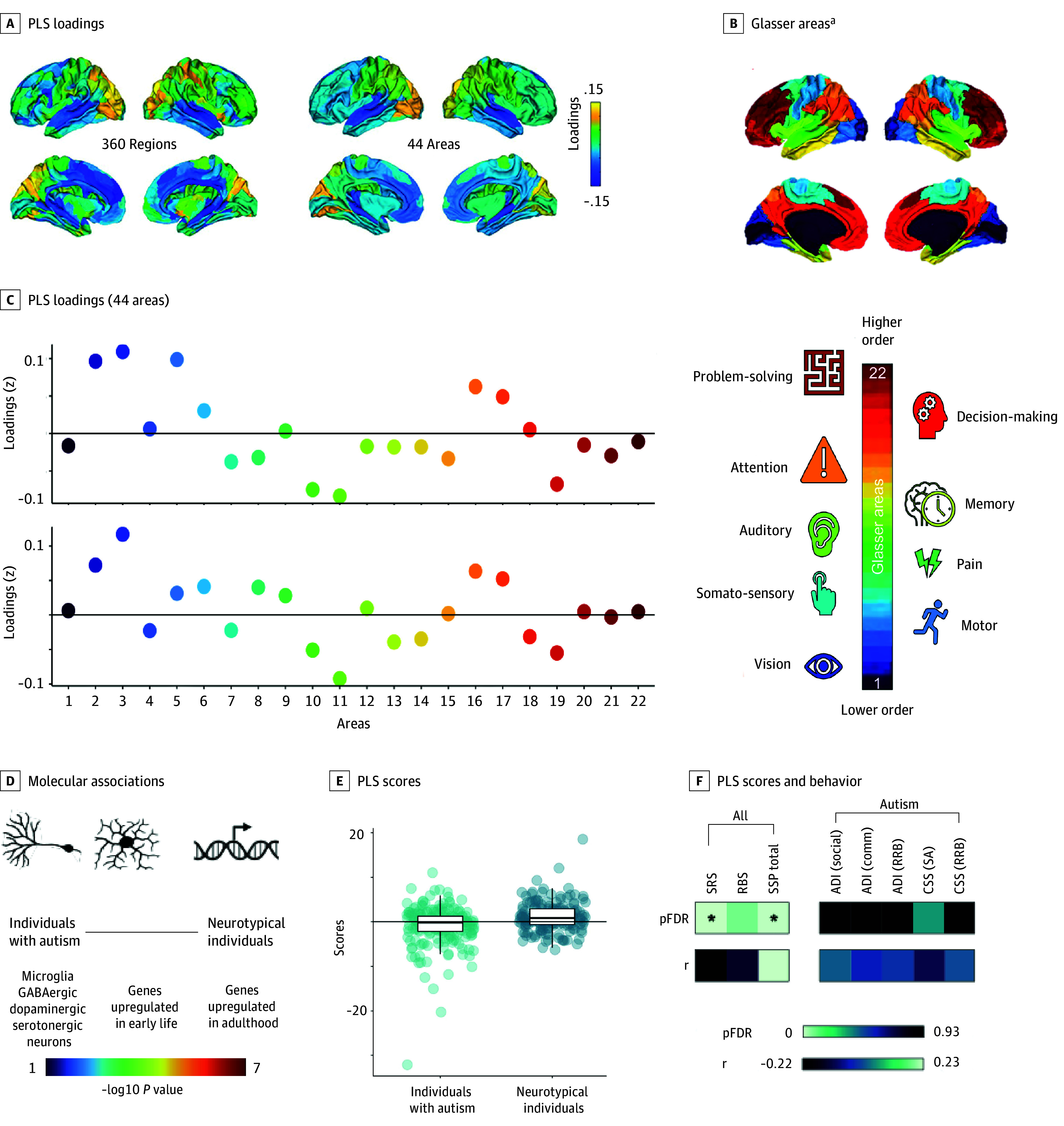
Spatial Patterns of Developmental Differences in Cortical Thickness and Their Molecular and Cognitive-Behavioral Correlates A, Partial least squares (PLS) loadings in 360 regions and 44 areas. B, Glasser areas and functional correlates (colors encode Glasser areas 1-22). C, Standardized PLS areal loadings (numbers represent Glasser areas). Interpretation: in areas with positive loadings, neurotypical individuals (vs autistic participants) display a more positive change (smaller decrease/larger increase); in areas with negative loadings, neurotypical individuals (vs autistic participants) display a more negative change (larger decrease/smaller increase) in cortical thickness. D, Molecular associations of spatial patterns along the continuum from more autistic to more neurotypical neuroanatomical development. Text color indicates significance of enrichment. E, PLS scores per group. F, Correlation between PLS scores and behavior. ADI indicates Autism Diagnostic Interview; comm, communication; CSS, Autism Diagnostic Observation Schedule calibrated severity score; LH, left hemisphere; pFDR, false discovery rate-corrected *P* values; RBS, Repetitive Behaviors Scale; RH, right hemisphere; RRB, Restricted and Repetitive Behaviors Subscale; SA, social affect; social, social domain; SRS, Social Responsiveness Scale; SSP, Short Sensory Profile. ^a^Glasser areas: 1, primary visual cortex (cx); 2, early visual cx; 3, dorsal stream visual cx; 4, ventral stream visual cx; 5, Mt plus; 6, somatosensory motor cx; 7, paracentral lobular and midcingulate cx; 8, premotor cx; 9, posterior opercular cx; 10, early auditory cx; 11, auditory association cx; 12, insular and frontal opercular cx; 13, medial temporal cx; 14, lateral temporal cx; 15, temporo-parieto-occipital junction; 16, superior parietal cx; 17, inferior parietal cx; 18, posterior cingulate cx; 19, anterior cingulate and medial prefrontal cx; 20, orbital and polar frontal cx; 21, inferior frontal cx; 22, dorsolateral prefrontal cx. The Mt plus area covers 9 visual areas in the lateral occipital and posterior temporal cortex.

**Figure 3.**
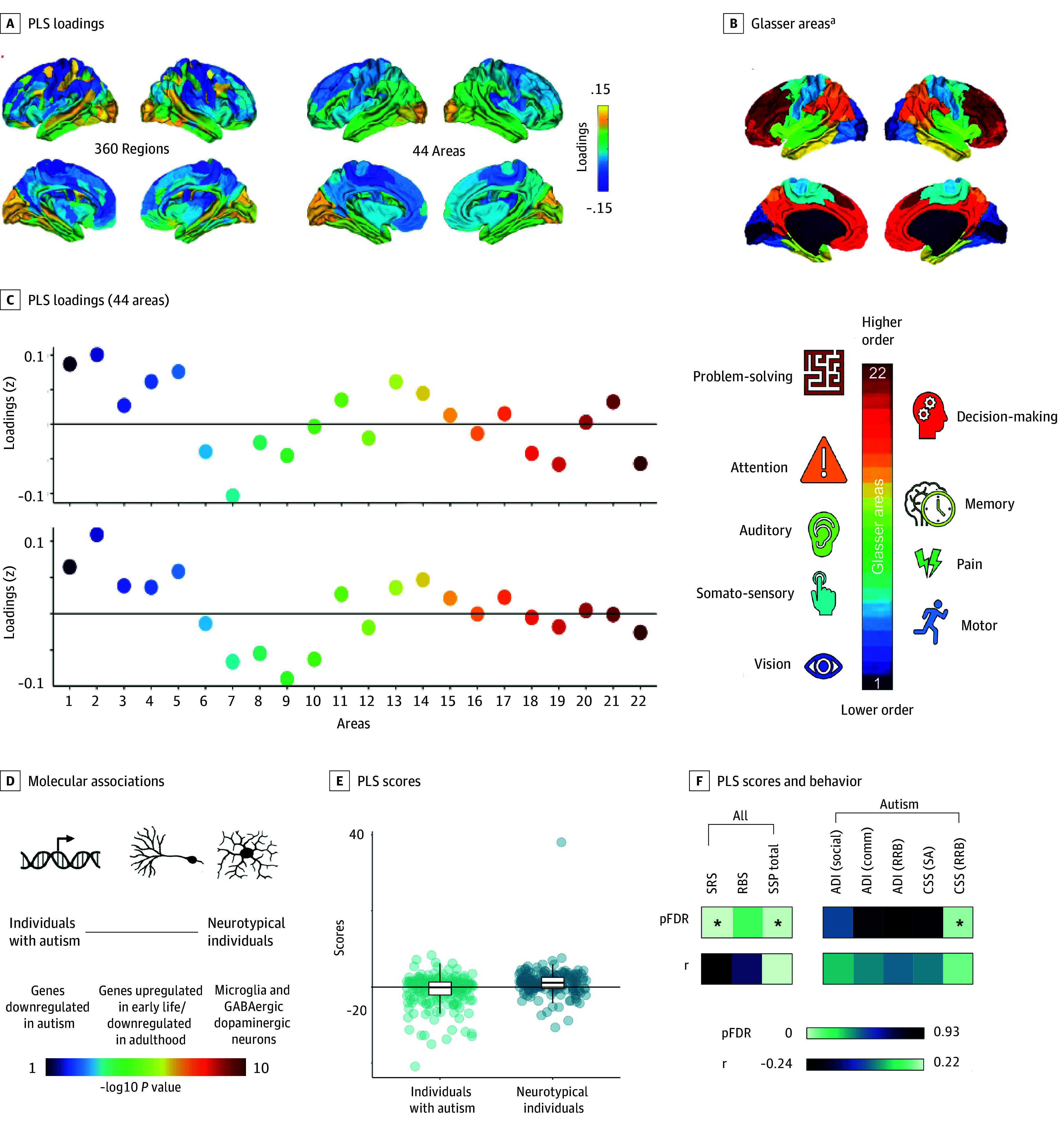
Spatial Patterns of Developmental Differences in Surface Area and Their Molecular and Cognitive-Behavioral Correlates A, Partial least squares (PLS) loadings in 360 regions and 44 areas. B, Glasser areas and functional correlates (colors encode Glasser areas 1-22). C, Standardized PLS areal loadings (numbers represent Glasser areas). Interpretation: in areas with positive loadings, neurotypical individuals (vs autistic participants) display a more positive change (smaller decrease/larger increase); in areas with negative loadings, neurotypical individuals (vs autistic participants) display a more negative change (larger decrease/smaller increase) in surface area. D, Molecular associations of spatial patterns along the continuum from more autistic to more neurotypical neuroanatomical development. Text color indicates significance of enrichment. E, PLS scores per group. F, Correlation between PLS scores and behavior. ADI indicates Autism Diagnostic Interview; comm, communication; CSS, Autism Diagnostic Observation Schedule calibrated severity score; LH, left hemisphere; pFDR, false discovery rate-corrected *P* values; RBS, Repetitive Behaviors Scale; RH, right hemisphere; RRB, Restricted and Repetitive Behaviors Subscale; SA, social affect; social, social domain; SRS, Social Responsiveness Scale; SSP, Short Sensory Profile. ^a^Glasser areas: 1, primary visual cortex (cx); 2, early visual cx; 3, dorsal stream visual cx; 4, ventral stream visual cx; 5, Mt plus; 6, somatosensory motor cx; 7, paracentral lobular and midcingulate cx; 8, premotor cx; 9, posterior opercular cx; 10, early auditory cx; 11, auditory association cx; 12, insular and frontal opercular cx; 13, medial temporal cx; 14, lateral temporal cx; 15, temporo-parieto-occipital junction; 16, superior parietal cx; 17, inferior parietal cx; 18, posterior cingulate cx; 19, anterior cingulate and medial prefrontal cx; 20, orbital and polar frontal cx; 21, inferior frontal cx; 22, dorsolateral prefrontal cx. The Mt plus area covers 9 visual areas in the lateral occipital and posterior temporal cortex.

We identified 1 differential component (ie, maturational pattern) per morphometric feature. The cortical thickness component was anchored in early auditory/auditory association and anterior cingulate cortex on one end (negative loadings) and in early/dorsal stream visual and parietal cortex on the other (positive loadings), with the remaining brain regions (eg, frontal cortex) contributing relatively little (Figure 2A-C). The surface area gradient stretched from the paracentral lobular, midcingulate, premotor, and posterior opercular cortex (negative loadings) to the primary/early visual and medial/lateral temporal cortex (positive loadings) with other regions (eg, parietal and orbital/polar frontal cortex) contributing little (Figure 3A-C). To facilitate the components’ visual interpretation, we grouped the 360 regions (180 per hemisphere) into 44 contiguous areas (22 per hemisphere)^18^ and displayed these areas’ average loadings on the cortical surface (Figure 2A and Figure 3A) and, as standardized values, in scatterplots (Figure 2C and Figure 3C).

#### Secondary Results

We replicated our results in an independent, external, longitudinal dataset (eFigures 1 and 2 in Supplement 1). Also, we retained our results when (1) repeating our analyses within individual age groups (eFigure 3 and eTable 2 in Supplement 1), (2) computing our analyses using an alternative cortical parcellation^21^ (eFigure 4A in Supplement 1), (3) accounting for the outcomes of medication (eFigure 4B in Supplement 1), and (4) conducting our analyses using continuous, rather than categorical, outcome variables related to autism, both within and across groups (eFigures 4C, 5, and 6 in Supplement 1).

### Genomic Results

#### Primary Results

The differential maturational patterns were associated with genes linked to various cell types, neurodevelopmental epochs/ages, and genes differentially expressed in autism.

Genes whose expression pattern was positively associated with a more neurotypical/less autismlike pattern were enriched for genes (1) linked to microglia and GABAergic and dopaminergic neurons (surface area) and (2) genes upregulated in adulthood (cortical thickness), upregulated in early life (surface area), and downregulated in adulthood (surface area) (eTable 3 in Supplement 1, Figure 2D and Figure 3D, and eFigures 7A, 7C, 8A, 8C, and 9 in Supplement 1).

Genes whose expression pattern was negatively associated with a more neurotypical/less autismlike pattern were enriched for genes (1) linked to microglia and dopaminergic, serotonergic, and GABAergic neurons (cortical thickness), (2) genes upregulated in early life (cortical thickness), and (3) downregulated in autism, including, eg, genes linked to synaptic development, chemical (neurotransmitter) and electric (calcium) synaptic transmission, and cell-cell signaling (surface area) (eTable 4 in Supplement 1, Figure 2D and Figure 3D, and eFigures 7B, 7D, 8B, and 8D in Supplement 1).

#### Secondary Results

Across features, genes positively associated with a more neurotypical/less autismlike pattern were enriched for genes regulating biological processes including cell cycle, cell growth, DNA metabolism and transcription, neuron development, and neurogenesis (eTable 5 in Supplement 1). In contrast, genes negatively associated with a more neurotypical/less autismlike pattern were enriched for genes regulating cell signaling, secretion, metabolism, and immune response (eTable 5 in Supplement 1).

### Cognitive Behavioral Results

#### Primary Results

Participants’ scores (ie, alignments with each component) (Figure 2E and Figure 3E) correlated with their cognitive behavioral profiles across both sets of evaluated measures. Across groups, participants with higher (ie, more neurotypical/less autismlike) scores in cortical thickness/surface area displayed fewer social difficulties (SRS) and more neurotypical sensory processing (SSP) (Figure 2F and Figure 3F and eFigures 11-14 in Supplement 1). In the autistic group, individuals with a more neurotypical/less autismlike profile had greater difficulties in repetitive behavior (for surface area only) (Figure 3F and eTables 6 and 12 in Supplement 1).

#### Secondary Results

Further, individuals with greater scores had significantly fewer difficulties in multiple sensory domains (SSP), including tactile, taste/smell (cortical thickness only), underresponsivity, auditory filtering, low energy (cortical thickness only), auditory sensitivity, hypersensitivity, and hyposensitivity (eFigure 10 in Supplement 1).

## Discussion

In this case-control study, we studied how brain regions develop in relation to each other in autistic individuals compared with neurotypical individuals and how differences correlate to molecular/genomic mechanisms and behavior. We established maturational between-group differences in cortical thickness and surface area that were mostly driven by sensorimotor regions and transcriptomically enriched for genes expressed in multiple cell types and neurodevelopmental stages and autism candidate genes. More neurotypical/less autismlike maturational profiles correlated with fewer social difficulties and more typical sensory processing. Results were replicated in an independent-collected cohort. Our study elucidates brain maturational differences and the associated complex interplay of temporally sensitive molecular mechanisms that may underpin both lower-order (eg, sensory processing) and higher-order (eg, social communication, restricted/repetitive behaviors) clinical features in autism.

### Neuroanatomy

In autistic individuals (vs neurotypical individuals), brain regions developed differently in relation to each other.

Across hemispheres and features, visual, auditory, and motor cortices contributed most, and prefrontal cortices least, to between-group differences. This corroborates previous reports of differential neuroanatomical development in autism in the temporal/occipital cortex but diverges from prior studies showing strong between-group differences across prefrontal lobes.^33,34,35,36^ These discrepancies may stem from methodological differences. For instance, we studied neurodevelopment from childhood to adulthood using longitudinal data, whereas previous studies have often examined narrower (predominantly younger) age groups and/or relied on cross-sectional data.^35,37,38,39,40^ Also, we identified patterns of correlated maturational differences, whereas previous studies have predominantly focused on developmental differences of individual brain regions.

Further, some brain areas (eg, visual cortex) contributed positively to the identified patterns. Here, a greater neuroanatomical increase/lower decrease over time could be interpreted as more neurotypical, ie, less autismlike. Others (eg, auditory cortex) contributed negatively. Here, a greater decrease/lower increase indicated a more neurotypical/less autismlike growth trajectory. This reinforces reports that, in neurotypical individuals, brain regions display varying growth trajectories, and in autism, these trajectories may be different but in multiple ways, eg, accelerated, delayed, decelerated, or otherwise altered.^33^

Cortical thickness and surface area patterns largely overlapped but also displayed regional differences (eg, the primary visual cortex contributed less to cortical thickness differences than it did to surface area differences). This matches prior evidence that cortical thickness and surface area have separate, region-specific developmental trajectories (possibly due to their distinct neurobiological origins).^41,42^

Our results were replicated in an independent, longitudinal dataset and robust against several potential confounds, including age group, cortical parcellation scheme, medication, and dimensional (vs categorical) clinical outcome measures.

Thus, our study extends previous evidence that neuroanatomical development in autism differed by reporting altered maturational patterns in brain, especially in sensorimotor cortices.

### Association With Molecular Mechanisms

The differential maturational patterns were transcriptomically enriched for various cell types, neurodevelopmental epochs/ages, and genes differentially expressed in autism.

Surface area and cortical thickness patterns were linked to dopaminergic/GABAergic neurons and microglia. This follows previous reports linking typical (or atypical) brain development to neuronal and glial cell types,^43,44,45,46^ and atypical (GABAergic) neurotransmission.^47,48,49,50,51,52,53^ It also aligns with our observations that patterns were enriched for genes regulating neuronal/glial processes, including neurogenesis and the immune response of the brain.

Genes associated with a neurotypicallike cortical thickness and surface area pattern were enriched for genes upregulated in adulthood and early life, respectively. This matches recent observations in more than 50 000 neurotypical individuals^54^ and suggests that transcription levels change across the life span to promote the development of distinct morphometric features. However, we detected the opposite in autismlike cortical thickness patterns, which were associated with genes upregulated in early life. This inverse activation of regulatory elements suggests that altered timing of transcription may contribute to divergent brain maturation in autism.

The autismlike surface area pattern was enriched for genes downregulated in autism, including those influencing synaptic development/signaling.^55^ Surface area may plausibly be affected by synaptic processes (eg, through long-term potentiation-induced synaptogenesis and spine outgrowth^56^) in both the autistic and nonautistic brain. The specificity of the observed enrichment to autism raises the question if autistic (vs neurotypical) brain maturation is associated with distinct neurobiological (eg, synaptic) mechanisms. Congruently, prior evidence in autism, rare genetic conditions linked to autism (eg, Fragile X), and autism rodent models (eg, *SHANK3* gene) has linked altered brain development to various genes, including those regulating synaptic development.^38,57,58^

Notably, we inferred gene expression using the Allen Human Brain Atlas, which is based on postmortem adult RNA-sequencing samples. However, genetic mechanisms occurring at much earlier developmental stages may also influence the observed neuroanatomical patterns.^54^ Future high-resolution gene expression maps in the developing brain may help explore these mechanisms.

Taken together, coordinated differences in neuroanatomical development in autism were genetically enriched for multiple neuronal and glial cell types, time-sensitive regulatory elements, and genes previously implicated in autism.

### Association With Cognition and Behavior

Across groups, individuals with more neurotypical/less autismlike maturational patterns had fewer social difficulties and more typical sensory processing. This association could reflect the fact that maturational patterns and clinical features both correlate with group (diagnosis). Alternatively, it may indicate a plausible neurobiological relationship between typical (or atypical) brain maturation and clinical profiles. In fact, previous studies in autism have found an association between altered neuroanatomical development in sensory/motor cortices with both sensory processing^59^ and social communication^60^ difficulties. Also, when we examined the association between brain maturation and these clinical features separately within each diagnostic group, we identified patterns that were correlated with those identified across groups, and this was true especially for those maturational patterns linked to sensory processing features (in autism) and social communication (in the neurotypicals). Further research is required to delineate the exact biological relationship between brain maturation and clinical variation at baseline, follow-up, and change in clinical profiles over time. Thus, we extend previous research into the link between neuroanatomy and clinical profiles by showing that altered maturational patterns in autism relate to core autism features.

### Implications

Cortical maturation progresses from lower-order unimodal primary cortices to higher-order transmodal association cortices,^7,8,9^ ensuring the availability of lower-order to higher-order functions.^23^ Hence, developmental differences in lower-order regions may affect lower-order functions and the subsequent maturation of higher-order regions and their functional correlates. Accordingly, maturational disruptions (driven especially by lower-order regions) correlated with both lower- and higher-order cognitive functions. Notably, these maturational differences in lower-order regions may, themselves, result from earlier experiences. For instance, sensorimotor processing differences during infancy may shape the bulk of cortical maturation occurring before 6 years (age of the first scan in our sample) and, thereby, affect developmental cascades and contribute to autism core symptoms.^61,62,63,64^ Hence, maturational differences (driven by genetic mechanisms and by the interplay between altered sensorimotor processing and genetics) may interrupt brain structural/functional developmental cascades and drive the coexistence of both lower- and higher-order clinical features, representing a potential key etiological mechanism in autism.

### Next Steps

Future studies should examine vulnerability and resilience to neurodevelopmental disruptions and associated clinical profiles, eg, through a recently established, large-scale research consortium focusing on risk/resilience in neurodevelopmental conditions.^65^ This may pave a way for strategies to reduce vulnerability, and boost resilience, during critical developmental periods to improve clinical outcomes in individuals who want interventions.

In addition, future research should investigate the specificity of maturational differences and associated clinical outcomes to (subgroups of) individuals with neurodevelopmental/mental health conditions and if an individual’s position on the neurotypical to autismlike gradient could inform stratification/subtyping approaches. Leveraging existing, heterogeneous cohorts,^66,67^ such research may inform the development of predictive/prognostic biomarkers in autism and related conditions.

### Strengths and Limitations

Our study’s strengths include the longitudinal design, including of one of the world’s largest/most heterogeneous autism datasets, statistical approach (ours is among the first studies investigating correlated patterns of differential maturation in autism), and the replication in an independent cohort. Nonetheless, we acknowledge several limitations. We examined development over 12 to 24 months (a rare opportunity to study atypical brain maturation repeatedly as comparable datasets are scarce). Nonetheless, to investigate maturation over longer time periods, we are currently testing our participants for a third time. Also, our analyses were restricted to the cortex, enabling us to compare our results with previous literature on cortical brain maturation and associated molecular/cognitive-behavioral variation in autism^5,17,25,68,69,70^ and cortical maturation patterns.^7,8,9^

## Conclusions

Results of this case-control study reveal that the coordinated development of brain regions was altered in autism, involved a complex interplay of temporally sensitive molecular mechanisms, and may be associated with both lower-order (eg, sensory) and higher-order (eg, social) clinical features of autism. Thus, examining maturational patterns may provide an analytic framework to study the neurobiological origins of clinical profiles in neurodevelopmental/mental health conditions. Future studies should expand our analyses to additional brain structures (eg, subcortex/cerebellum).

## Notes

Supplement 1.
eMethods.
**eFigure 1.** Spatial Patterns of Neuroanatomical Development That Differed Between Groups: Replication **eFigure 2.** Spatial Patterns of Neuroanatomical Development That Differed Between Groups: Association Between Original and Replication Results **eFigure 3.** Spatial Patterns of Neuroanatomical Development That Differed Between Groups Within Children, Adolescents, And Adults **eFigure 4.** Spatial Patterns of Neuroanatomical Development That Differed Between Groups When Accounting for Potential Confounders **eFigure 5.** Spatial Patterns of Neuroanatomical (Cortical Thickness) Development Linked to Dimensional Clinical Profiles Across and Within Diagnostic Groups **eFigure 6.** Spatial Patterns of Neuroanatomical (Surface Area) Development Linked to Dimensional Clinical Profiles Across and Within Diagnostic Groups **eFigure 7.** Spatial Patterns of Neuroanatomical Development That Differed Between Groups and Their Genetic Enrichment for Neurodevelopmental Epochs **eFigure 8.** Spatial Patterns of Neuroanatomical Development That Differed Between Groups and Their Genetic Enrichment for Neurodevelopmental Ages **eFigure 9.** Spatial Patterns of Neuroanatomical Development That Differed Between Groups and Their Genetic Enrichment for Each Other **eFigure 10.** Spatial Patterns of Neuroanatomical Development That Differed Between Groups and Their Association With Sensory Processing Domains **eFigure 11.** Spatial Patterns of Cortical Thickness Development That Differed Between Groups (Partial Least Squares Scores) and Their Association With Autism Traits **eFigure 12.** Spatial Patterns of Surface Area Development That Differed Between Groups (Partial Least Squares Scores) and Their Association With Autism Traits **eFigure 13.** Spatial Patterns of Cortical Thickness Development That Differed Between Groups (Partial Least Squares Scores) and Their Association With Sensory Processing Domains **eFigure 14.** Spatial Patterns of Surface Area Development That Differed Between Groups (Partial Least Squares Scores) and Their Association With Sensory Processing Domains **eFigure 15.** Cortical Thickness Maturation in the Left Hemisphere Over Time (Residuals) **Figure 16.** Cortical Thickness Maturation in the Right Hemisphere Over Time (Residuals) **Figure 17.** Surface Area Maturation in the Left Hemisphere Over Time (Residuals) **Figure 18.** Surface Area Maturation in the Right Hemisphere Over Time (Residuals) **Figure 19.** Cortical Thickness Maturation in the Left Hemisphere Over Time **Figure 20.** Cortical Thickness Maturation in the Right Hemisphere Over Time **Figure 21.** Surface Area Maturation in the Left Hemisphere Over Time **Figure 22.** Surface Area Maturation in the Right Hemisphere Over Time **Table 1.** Medication **Table 2.** Spatial Components Within Individual Age Groups **Table 3.** Genes Positively Associated With a Neurotypical Maturational Pattern: Enrichment for Cell Types and Genes Differentially Expressed in Autism **Table 4.** Genes Negatively Associated With a Neurotypical Maturational Pattern: Enrichment for Cell Types and Genes Differentially Expressed in Autism **Table 5.** Genes Positively and Negatively Associated With a Neurotypical Maturational Pattern: Enrichment For Gene Ontology (GO) Biological Processes **Table 6.** Association Between Maturational Patterns and Behavioral Measures **Table 7.** Demographics of the BrainMapASD Sample **Table 8.** Demographics of the LEAP AIMS Subsample Matched in Age to BrainMapASD **Table 9.** Comparison of Demographics Between the Original (LEAP) and the Replication (BrainMapASD) Sample
eReferences

Supplement 2. **eTable 10.** For Each Participant, for Each Glasser Region, This Includes the Average Cortical Thickness (Presented Separately for the Left and Right Hemisphere) **eTable 11.** For Each Participant, for Each Glasser Region, This Includes the Average Surface Area (Presented Separately for the Left and Right Hemisphere) **eTable 12.** Across Groups and Participants, for Each Glasser Area, This Includes the Average Loading (Presented Separately for Each Feature and Hemisphere) **eTable 13.** Across Groups and Participants, for Each Glasser Region, This Includes the Average Loading (Presented Separately for Each Feature and Each Hemisphere)

Supplement 3. **Nonauthor Collaborators.** EU-AIMS LEAP Group.

Supplement 4.
Data Sharing Statement.
