# Supplementary material for: Patterns of Brain Maturation in Autism and Their Molecular Associations
Source: JAMA Psychiatry. 2024 Oct 16;81(12):1253–64. doi: 10.1001/jamapsychiatry.2024.3194 (PMC11581727; doi:10.1001/jamapsychiatry.2024.3194)
Supplement: Supplement 1. — eMethods. eFigure 1. Spatial Patterns of Neuroanatomical Development That Differed Between Groups: Replication eFigure 2. Spatial Patterns of Neuroanatomical Development That Differed Between Groups: Association Between Original and Replication Results eFigure 3. Spatial Patterns of Neuroanatomical Development That Differed Between Groups Within Children, Adolescents, And Adults eFigure 4. Spatial Patterns of Neuroanatomical Development That Differed Between Groups When Accounting for Potential Confounders eFigure 5. Spatial Patterns of Neuroanatomical (Cortical Thickness) Development Linked to Dimensional Clinical Profiles Across and Within Diagnostic Groups eFigure 6. Spatial Patterns of Neuroanatomical (Surface Area) Development Linked to Dimensional Clinical Profiles Across and Within Diagnostic Groups eFigure 7. Spatial Patterns of Neuroanatomical Development That Differed Between Groups and Their Genetic Enrichment for Neurodevelopmental Epochs eFigure 8. Spatial Patterns of Neuroanatomical Development That Differed Between Groups and Their Genetic Enrichment for Neurodevelopmental Ages eFigure 9. Spatial Patterns of Neuroanatomical Development That Differed Between Groups and Their Genetic Enrichment for Each Other eFigure 10. Spatial Patterns of Neuroanatomical Development That Differed Between Groups and Their Association With Sensory Processing Domains eFigure 11. Spatial Patterns of Cortical Thickness Development That Differed Between Groups (Partial Least Squares Scores) and Their Association With Autism Traits eFigure 12. Spatial Patterns of Surface Area Development That Differed Between Groups (Partial Least Squares Scores) and Their Association With Autism Traits eFigure 13. Spatial Patterns of Cortical Thickness Development That Differed Between Groups (Partial Least Squares Scores) and Their Association With Sensory Processing Domains eFigure 14. Spatial Patterns of Surface Area Development That Differed Between Groups (Partial Least Squares Scores) [file jamapsychiatry-e243194-s001.pdf]

Supplementary Online Content

Pretzsch CM, Arenella M, Lerch JP, et al; EU-AIMS LEAP Group. Patterns of brain maturation in autism and their molecular associations. *JAMA Psychiatry*. Published online October 16, 2024. doi:10.1001/jamapsychiatry.2024.3194

eMethods.

eFigure 1. Spatial Patterns of Neuroanatomical Development That Differed Between Groups: Replication

eFigure 2. Spatial Patterns of Neuroanatomical Development That Differed Between Groups: Association Between Original and Replication Results

eFigure 3. Spatial Patterns of Neuroanatomical Development That Differed Between Groups Within Children, Adolescents, And Adults

eFigure 4. Spatial Patterns of Neuroanatomical Development That Differed Between Groups When Accounting for Potential Confounders

eFigure 5. Spatial Patterns of Neuroanatomical (Cortical Thickness) Development Linked to Dimensional Clinical Profiles Across and Within Diagnostic Groups

eFigure 6. Spatial Patterns of Neuroanatomical (Surface Area) Development Linked to Dimensional Clinical Profiles Across and Within Diagnostic Groups

eFigure 7. Spatial Patterns of Neuroanatomical Development That Differed Between Groups and Their Genetic Enrichment for Neurodevelopmental Epochs

eFigure 8. Spatial Patterns of Neuroanatomical Development That Differed Between Groups and Their Genetic Enrichment for Neurodevelopmental Ages

eFigure 9. Spatial Patterns of Neuroanatomical Development That Differed Between Groups and Their Genetic Enrichment for Each Other

eFigure 10. Spatial Patterns of Neuroanatomical Development That Differed Between Groups and Their Association With Sensory Processing Domains

eFigure 11. Spatial Patterns of Cortical Thickness Development That Differed Between Groups (Partial Least Squares Scores) and Their Association With Autism Traits

eFigure 12. Spatial Patterns of Surface Area Development That Differed Between Groups (Partial Least Squares Scores) and Their Association With Autism Traits

eFigure 13. Spatial Patterns of Cortical Thickness Development That Differed Between Groups (Partial Least Squares Scores) and Their Association With Sensory Processing Domains

©2024 Pretzsch CM et al. *JAMA Psychiatry*.

eFigure 14. Spatial Patterns of Surface Area Development That Differed Between Groups (Partial Least Squares Scores) and Their Association With Sensory Processing Domains

eFigure 15. Cortical Thickness Maturation in the Left Hemisphere Over Time (Residuals)

Figure 16. Cortical Thickness Maturation in the Right Hemisphere Over Time (Residuals)

Figure 17. Surface Area Maturation in the Left Hemisphere Over Time (Residuals)

Figure 18. Surface Area Maturation in the Right Hemisphere Over Time (Residuals)

Figure 19. Cortical Thickness Maturation in the Left Hemisphere Over Time

Figure 20. Cortical Thickness Maturation in the Right Hemisphere Over Time

Figure 21. Surface Area Maturation in the Left Hemisphere Over Time

Figure 22. Surface Area Maturation in the Right Hemisphere Over Time

Table 1. Medication

Table 2. Spatial Components Within Individual Age Groups

Table 3. Genes Positively Associated With a Neurotypical Maturational Pattern: Enrichment for Cell Types and Genes Differentially Expressed in Autism

Table 4. Genes Negatively Associated With a Neurotypical Maturational Pattern: Enrichment for Cell Types and Genes Differentially Expressed in Autism

Table 5. Genes Positively and Negatively Associated With a Neurotypical Maturational Pattern: Enrichment For Gene Ontology (GO) Biological Processes

Table 6. Association Between Maturational Patterns and Behavioral Measures

Table 7. Demographics of the BrainMapASD Sample

Table 8. Demographics of the LEAP AIMS Subsample Matched in Age to BrainMapASD

Table 9. Comparison of Demographics Between the Original (LEAP) and the Replication (BrainMapASD) Sample

eReferences

This supplementary material has been provided by the authors to give readers additional information about their work.

eMethods

Participants

We included male and female autistic and neurotypical participants aged 6-30 years (at the first visit) if they or their parents/guardians were able to provide informed written or verbal consent/assent to their participation in this study, and if they had a high-quality structural MRI scan. We included autistic participants if they had an existing clinical diagnosis of ASD in line with DSM-IV/ICD-10 or DSM-5 criteria[1,2]. As most autistic individuals have one or more co-occurring psychiatric conditions[3], all psychiatric comorbidities (except for psychosis and bipolar disorders) were allowed. Further, in light of the high number of autistic individuals who are being prescribed regular medication (30-50% in Europe[4] and 70% in the US[5]), we included participants on stable medication. In contrast, we excluded participants with conditions preventing them from safe scanning (e.g., metal objects in their body). The study was approved by national and local ethics review boards at all study sites. These included the London-Central and Queen Square Health Research Authority Research Ethics Committee (University of Cambridge and King's College London; ID 13/LO/1156), the UMM University Medical Mannheim Medical Ethics Commission II (Mannheim University; ID 2014-540N-MA), the Radboud University Medical Centre Institute Ensuring Quality and Safety Committee on Research Involving Human Subjects Arnhem-Nijmegen (Radboud University and Utrecht University; ID 2013/455), and the University Campus Bio-Medical Ethics Committee De Roma (Rome University; ID 18/14 PAR ComET CBM). This study was carried out according to Good Clinical Practice (ICH GCP) standards. The replication dataset was derived from the BrainMapASD cohort, an independent longitudinal study that was approved by the Ethics Committee of the Faculty of Medicine of Goethe University Frankfurt (approval number: 124/14; date of approval: 29th March 2016; for more information, see[6]).

Cortical reconstruction using FreeSurfer

We processed our images using the automated longitudinal stream[7] in FreeSurfer v6.0 (surfer.nmr.mgh.harvard.edu/). FreeSurfer uses robust, inverse consistent registration to create an unbiased within-subject template space (base image)[8]. Common information from this base image is then used to perform pre-processing, including e.g., skull stripping, transformation to Talairach space etc. This significantly increases reliability and statistical power (compared to using information from a single timepoint only)[7]. Using this pre-processed data, we reconstructed cortical surfaces via well-validated and fully automated procedures (e.g.,[9-12]). Surfaces were inspected visually for reconstruction errors (by three experienced researchers) and manually edited if needed. Following the exclusion of scans due to missing time points (e.g., no follow-up scans available), scanner upgrades, missing demographic information, or poor data quality, we retained 386 participants (214 autistic, 172 neurotypical).

We then computed vertex-wise measures and whole-brain averages (used as covariates in subsequent statistical analyses) of surface area (as outlined by Winkler et al.[13]) and cortical thickness (calculated as the closest distance from the grey-white matter boundary to the grey matter-cerebrospinal fluid boundary at each vertex on the tessellated surface[11]). Neuroanatomical development between T1 and T2 was quantified using FreeSurfer's "symmetrized percentage change" (SPC). The SPC captures the vertex-wise rate of change in feature X with respect to the average of feature X across time (T) points (T1 and T2)), i.e., $SPC = (100 \times \ [(X_{T2} - \ X_{T1}) \div (T_{T2} - T_{T1})]) \div [0.5 * (X_{T1} + \ X_{T2})]$.

©2024 Pretzsch CM et al. *JAMA Psychiatry*.

Previous studies suggest that the SPC is more robust and has greater statistical power when examining change between time points than commonly used alternative measures, such as the difference (T2-T1), ratio (T2/T1), or percent change (100*(T2-T1/T1))[14]. All features were smoothed (10 mm surface-based kernel) in accordance with previously published recommendations[15]. Given that our data was collected across multiple study centres, we applied ComBat batch effect harmonization[16] to account for site effects prior to statistical modelling.

Statistical analysis

Neuroanatomical analyses

Primary analyses

First, we removed the effects of potential confounders (age at T1 and its interaction with the follow-up duration, IQ, sex, and total brain measures [mean cortical thickness and total surface area, respectively]) from our neuroanatomical data using linear regression. The resulting residuals were down-sampled from >320k vertices across hemispheres to 360 regions across hemispheres (180 regions per hemisphere; based on the Glasser atlas[17]) to make our results more interpretable (including in the context of other work, such as the Human Connectome Project[18]) and computationally feasible. We selected the Glasser parcellation scheme because it is well-established, validated, based on multiple imaging modalities (unlike other commonly used unimodal atlases e.g.,[19-21]) and it balances spatial resolution with interpretability.

Next, to identify patterns of neuroanatomical developmental differences between autistic and neurotypical participants, we performed a partial least squares analysis (PLS) with a categorical dependent variable, aka partial least squares discriminant analysis (PLS-DA). Briefly, this analysis extracts a set of latent factors (components) that explain the maximum amount of covariance between the independent variable (i.e., neuroanatomical development in the parcellated brain regions) and the dependent variable (here: diagnostic group, with the reference group = neurotypicals). **While PLS-DA is frequently used as a machine learning algorithm for predictive performance, it can also be applied for explanatory purposes, i.e., to explore the association between the independent and dependent variable[22]; and this is what we did here. We chose to perform a PLS-DA analysis because, unlike other related statistical approaches (e.g., principal component analysis [PCA] and principal component regression [PCR]), PLS decomposes the input data in a supervised fashion, i.e., considering the dependent variable. As we were interested in identifying components that explain the greatest amount of variability in the dependent variable (diagnostic group), a PLS was more suitable than a PCA or PCR.** Here, we applied PLS-DA to the neuroanatomical data (360 datapoints or regions per person). We centred and scaled our data to improve numerical stability. Model parameters were tuned using cross-validation. Specifically, we selected leave-one-out cross validation, which is recommended for smaller sample sizes, and has been used in studies of participant samples like ours, e.g.,[23]. We identified the optimal number of components to explain group (for each morphometric feature), based on the largest value of the receiver operating characteristic (ROC).

©2024 Pretzsch CM et al. *JAMA Psychiatry*.

Interpretation of components
Broadly, the resulting components can be understood as spatial patterns of developmental differences between autistic and neurotypical individuals, i.e., those regions which, together, develop most differently in autistic vs neurotypical participants. Component scores and loadings indicate how each participant and brain region relates to these components, respectively. High absolute values indicate a strong correlation, while low absolute values show a weak correlation. Scores, ranging from negative to positive, indicate where an individual falls on the gradient from most autism-like/least neurotypical to least autism-like/most neurotypical (in terms of brain maturational profile). Loadings also range from negative to positive. In regions with negative loadings, neurotypicals display a more 'negative' neuroanatomical change (i.e., either a larger decrease or smaller increase in a feature) compared to the autistic individuals. In regions with positive loadings, neurotypicals show a more 'positive' change (i.e., either a smaller decrease or larger increase in a feature) compared to the autistic group. Individuals and regions with similar scores or loadings occupy nearby positions in a component (irrespective of their true location in the cortex). They can be considered similar with respect to a given property (e.g., their atypical developmental trajectory across age), while those at opposing ends of a component are maximally different regarding said property[24].

Secondary analyses

We performed secondary analyses to establish the robustness of our results.

First, we tested the replicability of our results using an independently collected cohort of autistic and neurotypical individuals. Briefly, this sample (called BrainMapASD) included longitudinal neuroimaging data from 146 children and adolescents aged 11-18 years (49 autistic, 97 neurotypical; 46 females, 100 males). We compared our original results to those obtained when repeating our analyses in BrainMapASD. Also, given the different age-ranges of LEAP and BrainMapASD, we repeated our analyses in a subset of LEAP that matched the age-range of BrainMapASD; and then compared these results to those obtained in BrainMapASD.

Second, as autism is a neurodevelopmental condition in which the association between brain and behaviour may vary across age[25,26], we repeated our analyses within each individual age group. We examined children (6-11 years; n=76; 33 autism, 43 neurotypical), adolescents (12-17 years; n=146; 84 autism, 62 neurotypical), and adults (18-30 years, n=164; 97 autism, 67 neurotypical). To further explore the effect of age-group, we also examined the spatial correlation between components identified across age-groups and those identified within age-groups.

Third, given reports that the varying spatial coarseness of different brain parcellations may influence observed neuroanatomical between-group differences[27], we repeated our analyses using an alternative cortical parcellation atlas. Instead of the multimodal, high-resolution Glasser Atlas, we used the uni-modal, lower-resolution (148 regions across hemispheres) Destrieux Atlas[20].

Fourth, while recent large-scale studies have reported no effect of medication on neuroanatomy in autism[28], we repeated our analyses while correcting for medication (no/unknown medication vs confirmed medication at time of first visit).

©2024 Pretzsch CM et al. *JAMA Psychiatry*.

Fifth, as autism is increasingly understood as a spectrum of abilities (rather than a binary outcome), we repeated our analyses to identify differential spatial patterns associated with dimensional cognitive-behavioural measures linked to autism (rather than using the categorical diagnosis as dependent variable). As we were interested in how cognitive-behavioural variation at baseline is associated with variance in subsequent brain development, we focused on clinical measures at T1. Specifically, we conducted separate PLS analyses for (i) the social responsiveness scale-22 (SRS-2)[29], (ii) the repetitive behaviour scale-revised (RBS-R)[30], and (iii) the short sensory profile (SSP)[31]. We selected these measures because they capture autism core- and associated features (i.e., social communication, restricted and repetitive behaviours, and sensory processing differences) and, unlike autism diagnostic measures, were obtained in both neurotypical and autistic participants. We examined the neuroanatomical patterns associated with these cognitive-behavioural measures both across and within diagnostic groups. The latter enabled us to better understand whether the dimensional brain-behaviour relationships observed across groups were due to the fact that behavioural profiles are correlated with diagnostic group; or whether they held within diagnostic groups.

Replication

Replication Methods

We replicated our results in the BrainMapASD dataset, which includes longitudinal structural magnetic resonance imaging and cognitive-behavioural measures from 146 children and adolescents with and without autism and was acquired in Frankfurt, Germany. A detailed description of this sample and its acquisition has previously been published in[6]. Please see below for a summary.

Procedure: Participants were included in BrainMapASD if they or their guardians (if participant age < 18 years) were able to provide informed written consent to their participation in this study and if they had a high-quality structural scan. Potential participants were excluded if they had contraindications to undergoing MRI scanning (e.g., metal in body), a history of major psychiatric or developmental conditions (e.g., psychosis), a head injury, genetic syndromes associated with autism (e.g., fragile-X syndrome), any medical condition affecting brain morphometry and function, or a history of drug abuse (e.g., alcohol). Autistic participants were included based on ADI-R[32] cut-offs (social: ≥ 10, communication ≥ 8, restricted and repetitive behaviours: ≥ 3; tolerance of -1 on the social/communication scale OR of -2 on the restricted and repetitive behaviours scale). Given the high number of autistic individuals who are being prescribed regular medication (30-50% in Europe[4] and 70% in the US[5]), participants on stable medication were included. Participants were scanned at two time points separated by ~2 years.

Data acquisition: BrainMapASD used 3T MRI (Siemens Magnetom Trio, MPRAGE ADNI sequence) to acquire high-resolution T1-weighted volumetric structural images at the Brain Imaging Centre Frankfurt (full head coverage, field of view=26.5cm, slice thickness=1mm, in-plane resolution 1*1mm^2, TR=2.3s, TE=2.2ms, FA=9°,176 slices).

Data processing: BrainMapASD data were processed and analysed congruent with the primary (LEAP AIMS) analyses, i.e., using the longitudinal stream[7] in FreeSurfer v6.0 (surfer.nmr.mgh.harvard.edu/) and following visual examination and manual edits (where required) by three independent raters.

Data analysis: To examine the replicability of our results, we performed two sets of analyses. First, we repeated our analyses in BrainMapASD; and compared our primary results to those obtained

in BrainMapASD. Second, given the different age-ranges between our primary sample and BrainMapASD, we repeated our analyses in a subset of LEAP (n=185) that matched the age-range of BrainMapASD (11-18 years); and then compared those results to the results obtained in BrainMapASD.

Replication Results

Demographics: The final sample included in this analysis comprised 146 participants (49 autistic and 97 neurotypical) children and adolescents (11-18 years). Groups did not differ significantly in age at T1 or T2, the time between visits (follow-up duration), FSIQ, mean cortical thickness, or total surface area. However, groups differed in sex (greater proportion males vs females in autism compared to neurotypicals) and autism symptom measures (as expected).

The subset of LEAP that was matched in age to the BrainMapASD sample included 185 participants (104 autistic, 81 neurotypical). Groups did not differ significantly in age at T1 or T2, the time between visits, mean cortical thickness, and total surface area. However, groups differed in FSIQ (higher in neurotypicals, as expected), sex (a greater proportion of males vs females in autism compared to neurotypicals), and autism symptomatology, as expected.

Neuroanatomical results in BrainMapASD: We identified one component that differentiated between neurotypical and autistic individuals for cortical thickness; and two components for surface area. These components resembled those identified in our primary analyses. Specifically, in line with our primary results, the cortical thickness component showed strong negative loadings in auditory cortices and positive loadings in visual cortex. Also, similar to our primary results, surface component 1 showed strong negative loadings in motor cortex and positive loadings in temporal regions; while surface area component 2 showed strong (albeit positive) loadings in premotor cortex, and (negative) loadings in temporal and frontal cortex.

Neuroanatomical results in age-matched LEAP sample: We identified two components for cortical thickness and one component for surface area. The cortical thickness component 1 was anchored in visual and motor cortex (positive loadings) and paracentral lobular, mid cingulate, anterior cingulate, and medial prefrontal cortex (negative loadings). The cortical thickness component 2 was anchored in posterior opercular cortex (negative loadings) and lateral temporal cortex and temporo-parieto-occipital junction (positive loadings). Across components, the strong loadings of visual, anterior cingulate, and parietal cortices resembled those observed in the BrainMapASD sample. The surface area component was anchored in primary visual cortex (positive loading), paracentral lobular and mid cingulate cortex (negative loading), medial temporal cortex (positive loading) and dorsolateral prefrontal cortex (positive loading). The strong premotor and temporal loadings resembled those observed in the BrainMapASD sample.

To facilitate the comparison between our primary results and those obtained in both the BrainMapASD and age-matched LEAP sample, we have plotted all results on the cortical surface (180 regional loadings per hemisphere) and as scatterplots (22 contiguous area loadings per hemisphere, as suggested by[17]). Also, we have correlated our primary and replication results within and across hemispheres using both regional (180/hemisphere) and areal (22/hemisphere) loadings.

©2024 Pretzsch CM et al. *JAMA Psychiatry*.

Genomic analyses

Gene expression decoding

To decode our neuroanatomical patterns, we leveraged the Allen Human Brain Atlas (AHBA)[33] of gene expression in human brain. First, we pre-processed the AHBA using the Abagen Toolbox (V0.1.1; github.com/rmarkello/abagen). Specifically, we re-annotated and intensity-filtered the microarray probes, normalized their coordinates, and allocated probes to cortical labels. We also normalized and rescaled expression values across genes and then across donor samples. This yielded a normalized expression matrix of 1,670 samples and 15,633 genes across donors with expression values ranging from 0 to 1. Pre-processed AHBA samples were mapped to the fsaverage6 surface (https://surfer.nmr.mgh.harvard.edu/fswiki/CoordinateSystems).

Next, we tested the spatial correlation between the mRNA expression patterns of the pre-processed AHBA genes and our neuroanatomical patterns. In line with[34], we fitted a Linear Mixed Effects (LME) model with a random intercept and slope grouped by donor to each gene's expression pattern to predict our neuroimaging map using the 'lme4' package in R (V1.1.28). This approach, described further in Ecker et al., under review, highlighted those genes whose expression pattern were consistently (i.e., across donors) similar to the input imaging map. Given the sparsity of samples within the AHBA/per donor, and our focus on identifying consistently high correlations across donors, we did not correct for spatial (auto-)correlations. We thresholded the resulting list of genes ($p_{FDR}<0.01$) to select an initial gene set for the subsequent enrichment analyses. Both sides of our imaging contrasts were of equal relevance, hence we considered both positive and negative t-statistic values. The resulting gene numbers were as follows: CT: $n_{pos}=291$, $n_{neg}=541$; SA: $n_{pos}=332$, $n_{neg}=267$.

Enrichment

Primary analyses

We examined the enrichment of the identified genes for various cell-types, neurodevelopmental epochs and ages, and genes differentially expressed in autism. We conducted separate enrichment analyses for those genes whose expression patterns correlated positively, and negatively, with the neuroanatomical components for each feature; and corrected our results for multiple comparisons (within features/gene sets) using FDR-correction ($p_{FDR}<0.05$).

We tested enrichment for cell-types using gene lists previously linked to neuronal and glial cell types in cortex[35] and the developing midbrain[36], and the GeneOverlap R package (V 1.36.0). To explore enrichment for neurodevelopmental epochs and ages, we conducted a hypothesis-free test of all genes annotated in the BrainSpan atlas of the developing human brain (brainspan.org) using functional Mapping and Annotation of Genome-Wide Association Studies (FUMA; fuma.ctglab.nl). Last, to examine enrichment for differentially expressed genes, we leveraged lists of genes that were up- or downregulated in post-mortem frontal and temporal cortex tissue of autistic individuals[37] and GeneOverlap.

Secondary analyses
To better understand the roles and functions of the identified genes, we also conducted hypothesis-free enrichment analyses of all biological processes annotated in the Gene Ontology Resource (geneontology.org) using gene set enrichment analysis within the Molecular Signatures Database (MSigDB) database (gsea-msigdb.org/gsea/msigdb). Moreover, to test how similar the genes linked to each neurodevelopmental pattern were to each other, we examined their mutual enrichment via GeneOverlap.

Cognitive-behavioural analyses

Primary analyses
Last, we examined how the neuroanatomical patterns identified above related to participants' cognitive-behavioural profiles at baseline.

To this aim, we correlated participants' scores, i.e., their alignment with each neuroanatomical pattern, with two sets of clinical measures. Set one included the SRS-2[29], RBS-R[30], and SSP[31]. As mentioned above, we selected these features because of their relevance to autism and their availability across groups. Set two included the autism diagnostic interview-revised (ADI-R)[32] and the autism diagnostic observation schedule (ADOS)[38,39]. We selected these measures because they represent so-called 'gold standard' instruments to assess autism symptom severity across core domains[40]. For the ADOS, we computed calibrated severity scores for social affect and restricted and repetitive behaviours to obtain standardized autism severity measures that account for differences in administered ADOS modules. We corrected our results for multiple comparisons across measures ($p_{FDR}<.05$).

Secondary analyses
Given that our neuroanatomical results were strongly driven by sensorimotor regions, we explored the association between neuroanatomy and different aspects of sensory processing in more detail. Specifically, we tested the correlation between participants' scores and the SSP[31] subdomains: tactile, taste/smell, movement, and visual/auditory sensitivity, under-responsivity/sensation seeking, auditory filtering, low energy/weakness, and composite measures of hyper- and hyposensitivity.

Genomic results

Secondary results

Genes associated positively with the more neurotypical/less autism-like cortical thickness component and negatively with the surface area component were significantly enriched for each other (Figure S9).

©2024 Pretzsch CM et al. *JAMA Psychiatry*.

eFigures

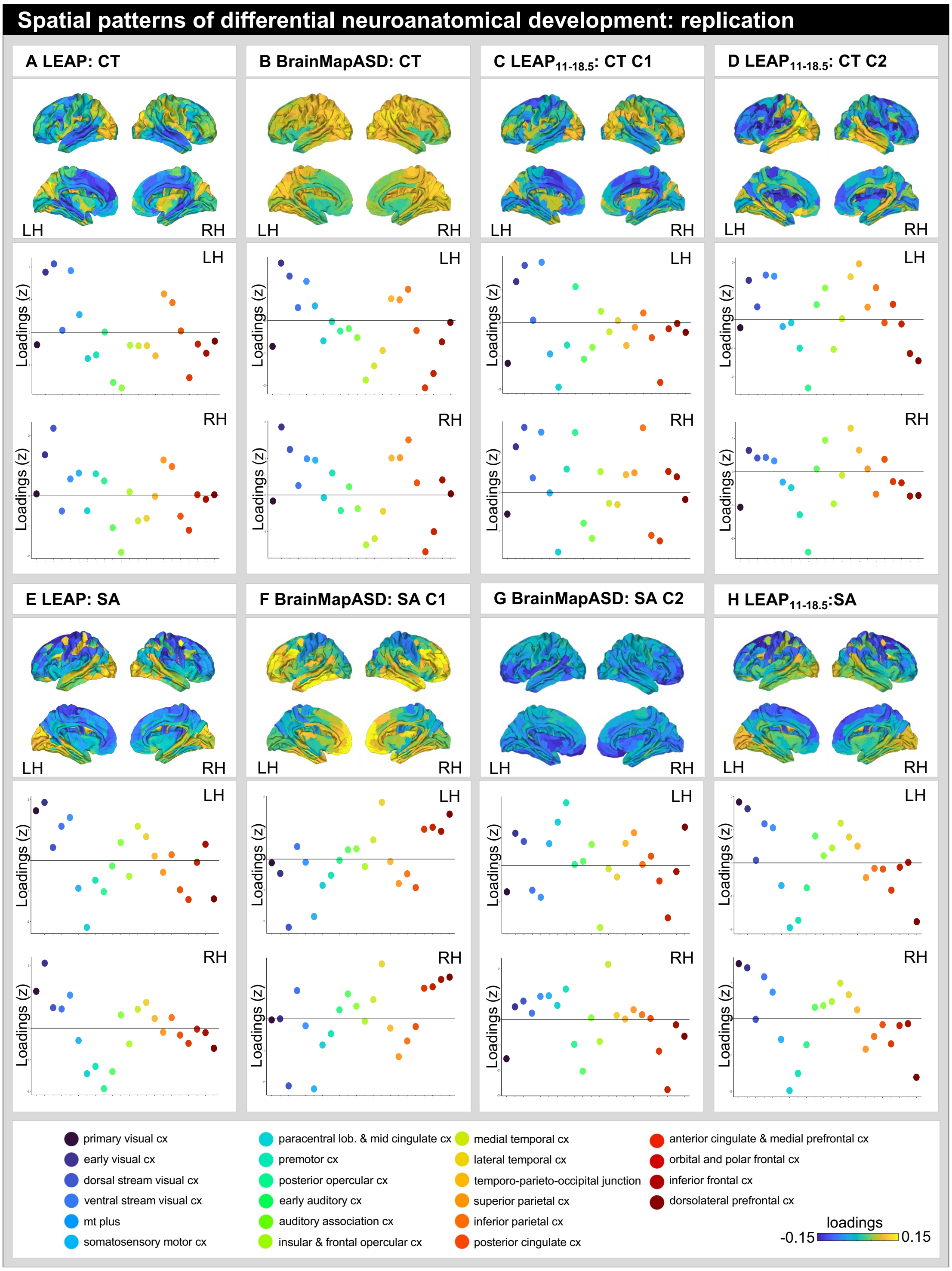

Figure S1 Spatial patterns of neuroanatomical development that differed between groups: replication. Visualization of 360 regional loadings on the cortical surface and 44 areal loadings (standardized) as scatterplots. A: LEAP sample, cortical thickness (CT) component. B: BrainMapASD, CT component. C: LEAP age-matched subsample CT component 1. D: LEAP age-matched subsample CT component 2. E: LEAP surface area (SA) component 1. F: BrainMapASD SA component 1. G: BrainMapASD SA component 2. H: LEAP age-matched subsample SA. The Colorbar indicates regional loadings in the brain maps. The named color legend at the bottom indicates the areal labels. Abbreviations: C, component/spatial pattern; CT, cortical thickness; CX, cortex; LH, left hemisphere; RH, right hemisphere; SA, surface area.

©2024 Pretzsch CM et al. *JAMA Psychiatry*.

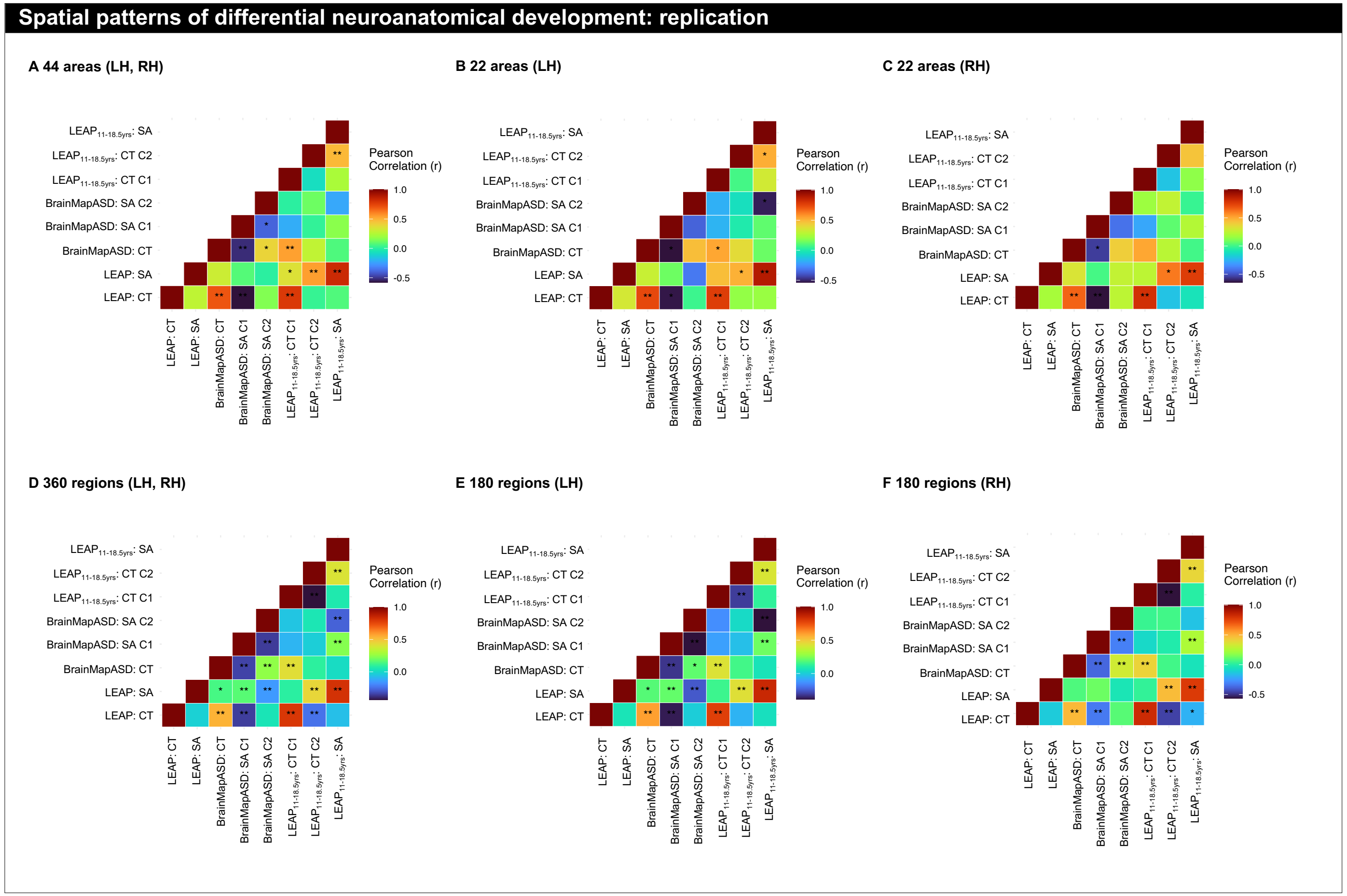

Figure S2 Spatial patterns of neuroanatomical development that differed between groups: association between original and replication results. Heatmaps indicate correlations between results obtained in the original sample (LEAP), the replication sample (BrainMapASD), and the age-matched subset of the original sample obtained using loadings of A: 44 areas across hemispheres, B: 22 areas in the left hemisphere, C: 22 areas in the right hemisphere; and loadings of D: 360 Glasser regions across hemispheres, E: 180 regions in the left hemisphere, and F: 180 regions in the right hemisphere. Colorbars indicate strength of association (Pearson Correlation coefficient, r). Stars on tiles indicate significance levels: "0:0.01 = '**'; 0.01:0.05 = '*'. Abbreviations: LH, left hemisphere; RH, right hemisphere.

©2024 Pretzsch CM et al. *JAMA Psychiatry*.

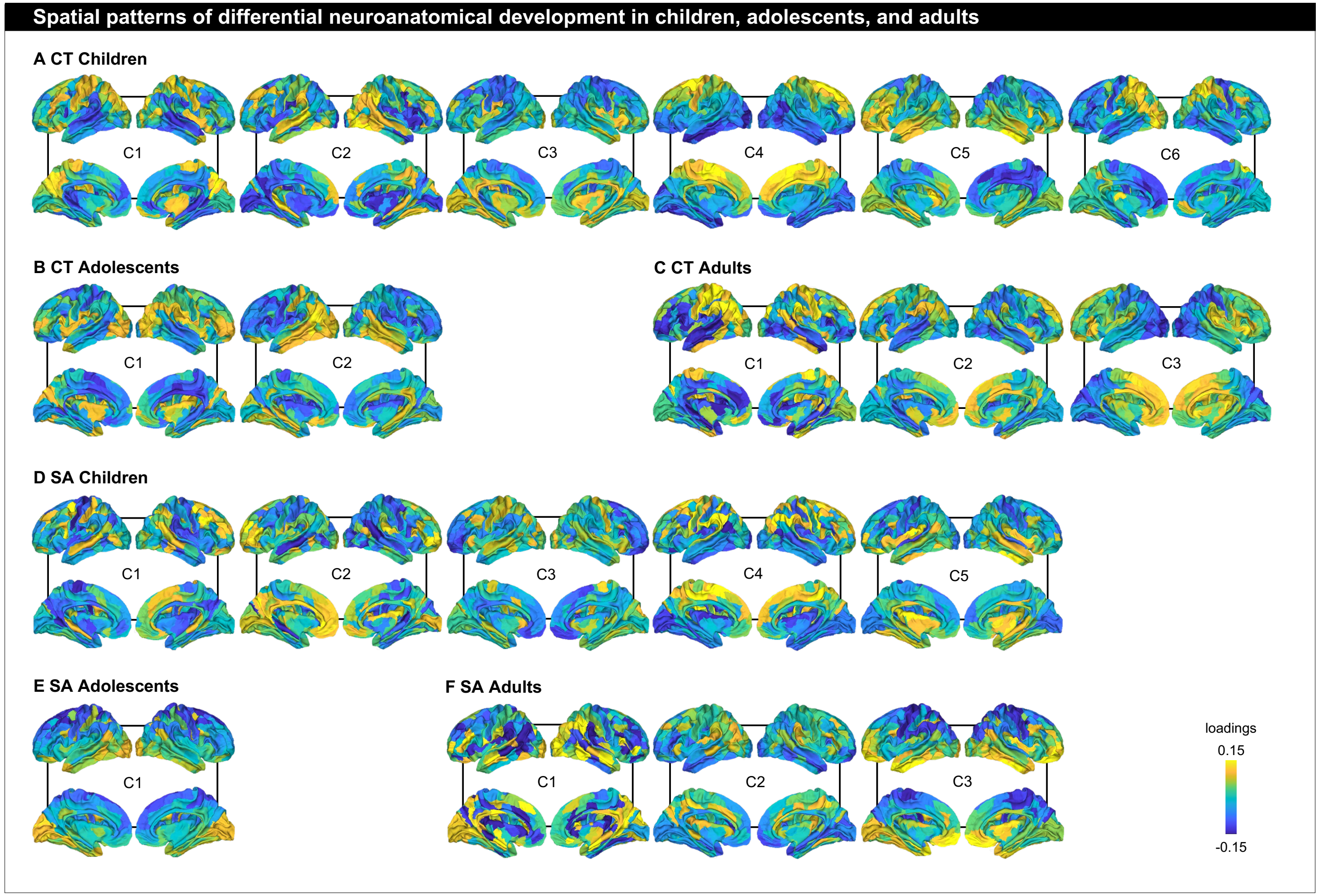

Figure S3 Spatial patterns of neuroanatomical development that differed between groups within children, adolescents, and adults. Visualization of 360 regional loadings on the cortical surface for cortical thickness (CT) in A: children, B: adolescents, and C: adults; and for surface area (SA) in D: children, E: adolescents, and F: adults. The colorbar indicates regional loadings onto components.

©2024 Pretzsch CM et al. *JAMA Psychiatry*.

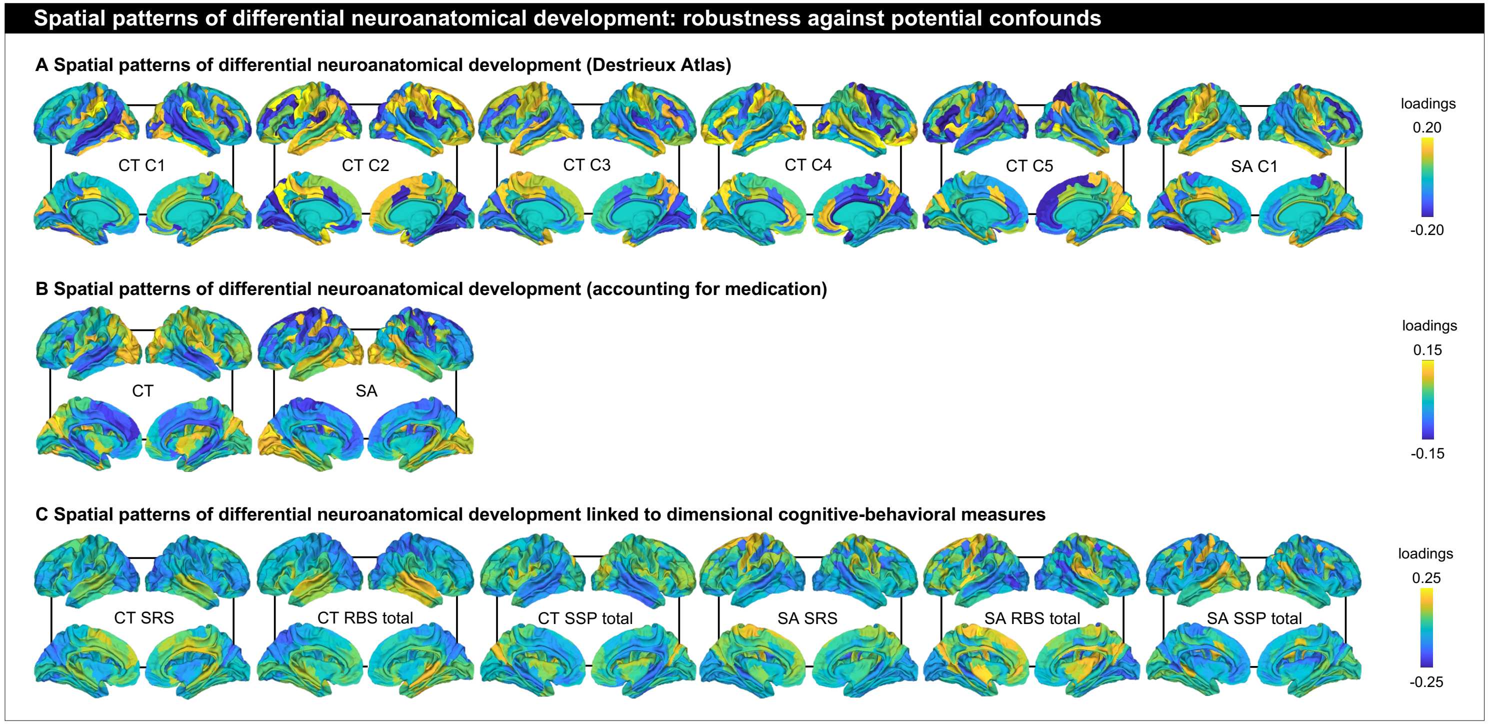

Figure S4 Spatial patterns of neuroanatomical development that differed between groups when accounting for potential confounders. A: using an alternative parcellation (Destrieux Atlas) to identify cortical thickness surface area patterns; B: correcting for medication to identify cortical thickness and surface area patterns; and C: using dimensional cognitive-behavioural features to study cortical thickness patterns linked to processing, repetitive behaviours, and sensory processing; and surface area patterns linked to social processing, repetitive behaviours, and sensory processing. The colorbars indicate regional loadings onto components. Abbreviations: CT, cortical thickness; RBS, repetitive behaviours scale; SA, surface area; SRS, social responsiveness scale; SSP, short sensory profile.

©2024 Pretzsch CM et al. *JAMA Psychiatry*.

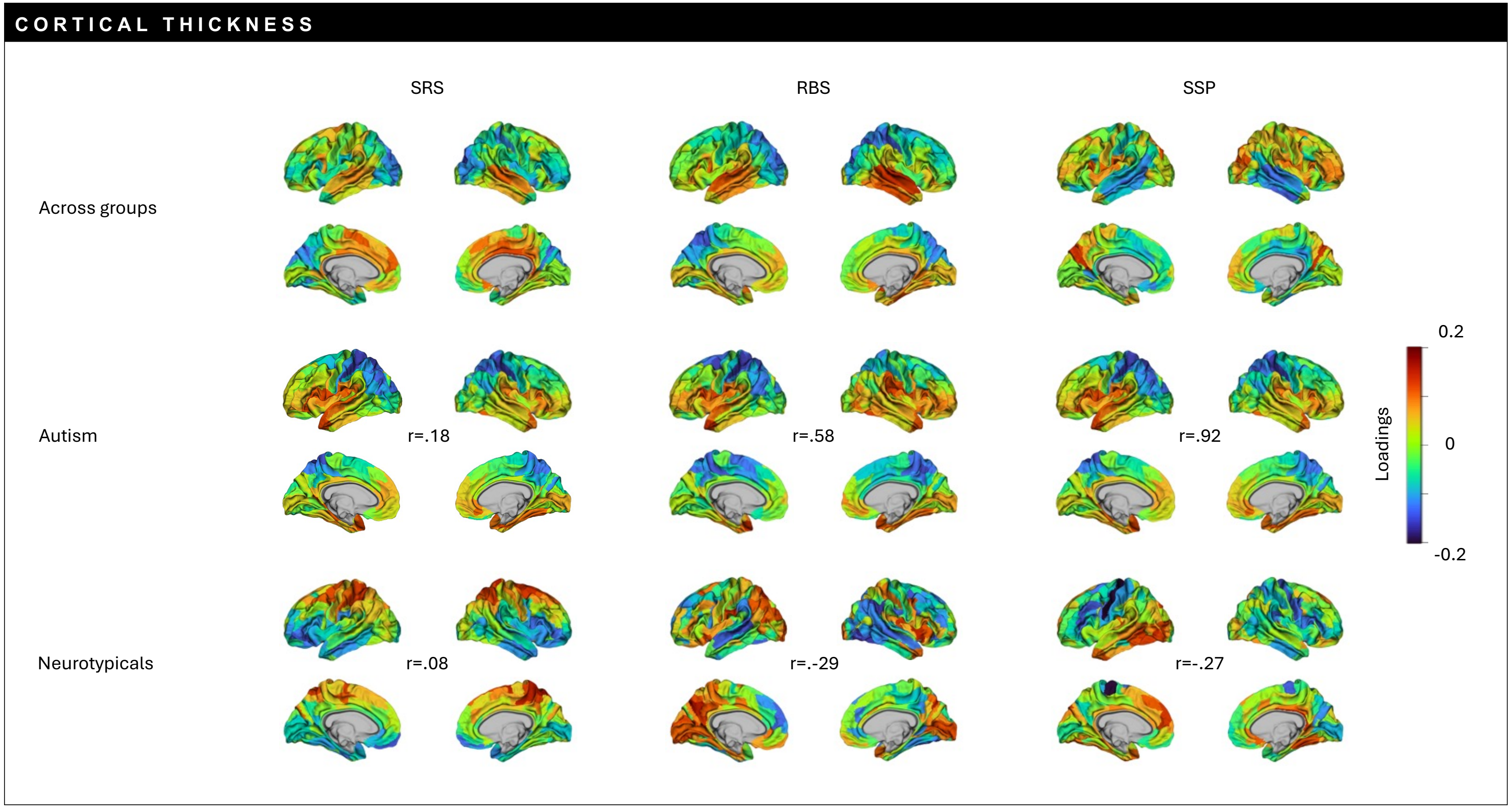

Figure S5 Spatial patterns of neuroanatomical (cortical thickness) development linked to dimensional clinical profiles across and within diagnostic groups. Correlation coefficients denote the Pearson correlation between the respective pattern identified within diagnostic group and the pattern identified across groups (top row). The colorbars indicate regional loadings onto components. Abbreviations: RBS, repetitive behaviours scale; SRS, social responsiveness scale; SSP, short sensory profile.

©2024 Pretzsch CM et al. *JAMA Psychiatry*.

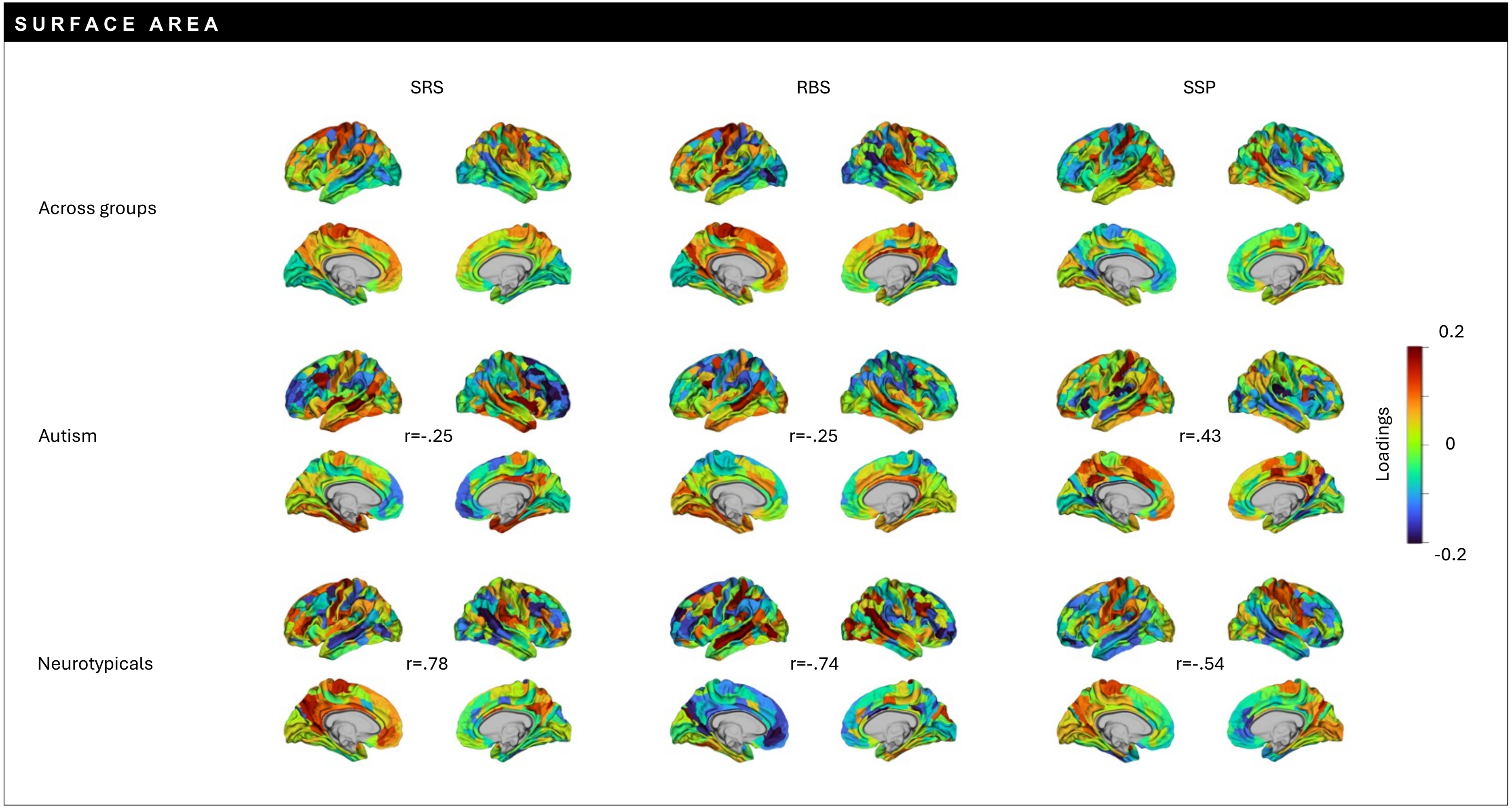

Figure S6 Spatial patterns of neuroanatomical (surface area) development linked to dimensional clinical profiles across and within diagnostic groups. Correlation coefficients denote the Pearson correlation between the respective pattern identified within diagnostic group and the pattern identified across groups (top row). The colorbars indicate regional loadings onto components. Abbreviations: RBS, repetitive behaviours scale; SRS, social responsiveness scale; SSP, short sensory profile.

©2024 Pretzsch CM et al. *JAMA Psychiatry*.

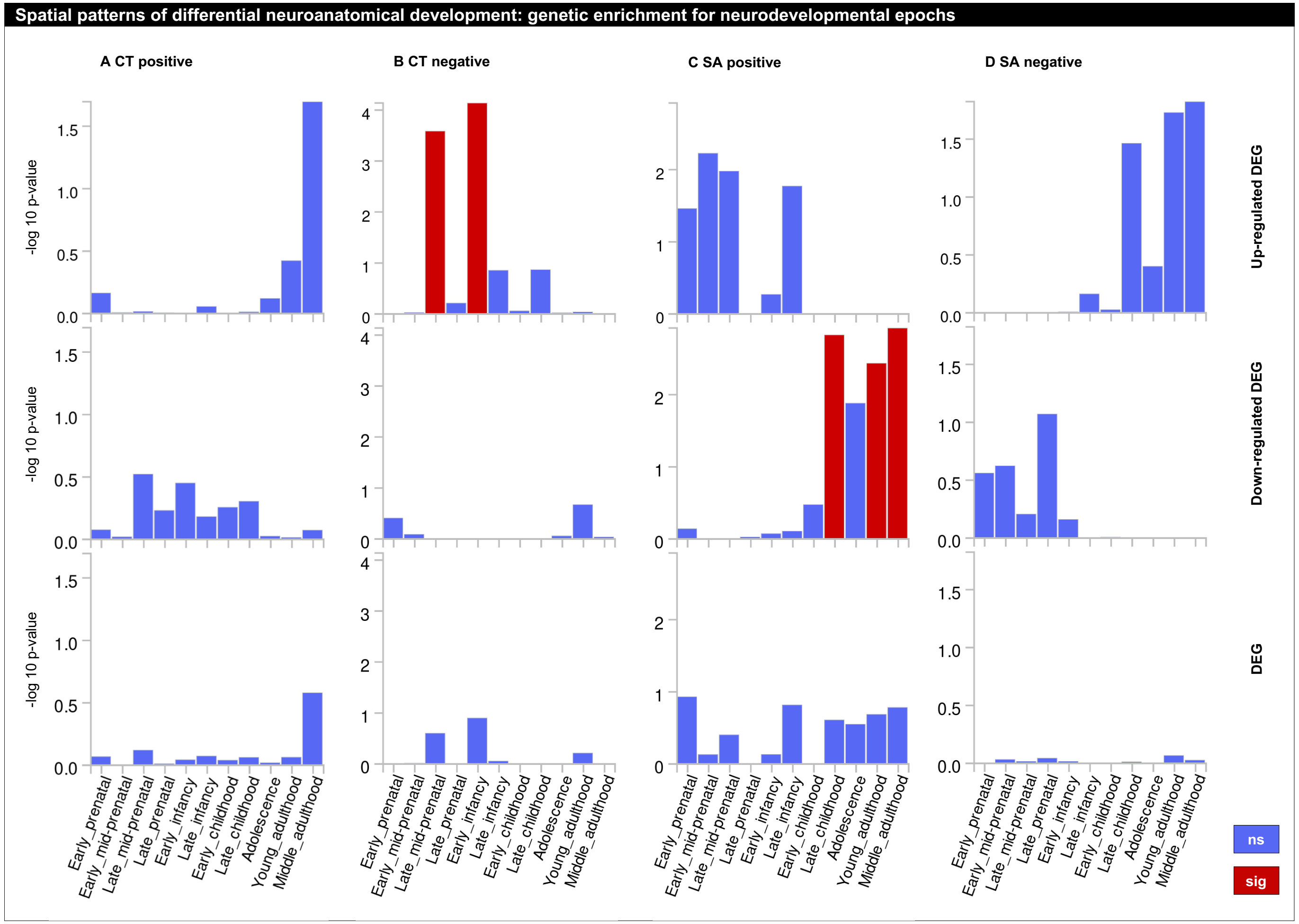

Figure S7 Spatial patterns of neuroanatomical development that differed between groups and their genetic enrichment for neurodevelopmental epochs. Association between genes positively and negatively associated with a more neurotypical/less autism-like neuroanatomical pattern in A: cortical thickness (CT), positive association, B: cortical thickness, negative association, C: surface area (SA), positive association, and D: SA, negative association. Each panel shows enrichment for developmental epochs. X-axes indicate epochs, and y-axes show significance levels (-log 10 p-values). Abbreviations: CT, cortical thickness; DEG, differential gene expression; ns, non-significant; sig, significant.

©2024 Pretzsch CM et al. *JAMA Psychiatry*.

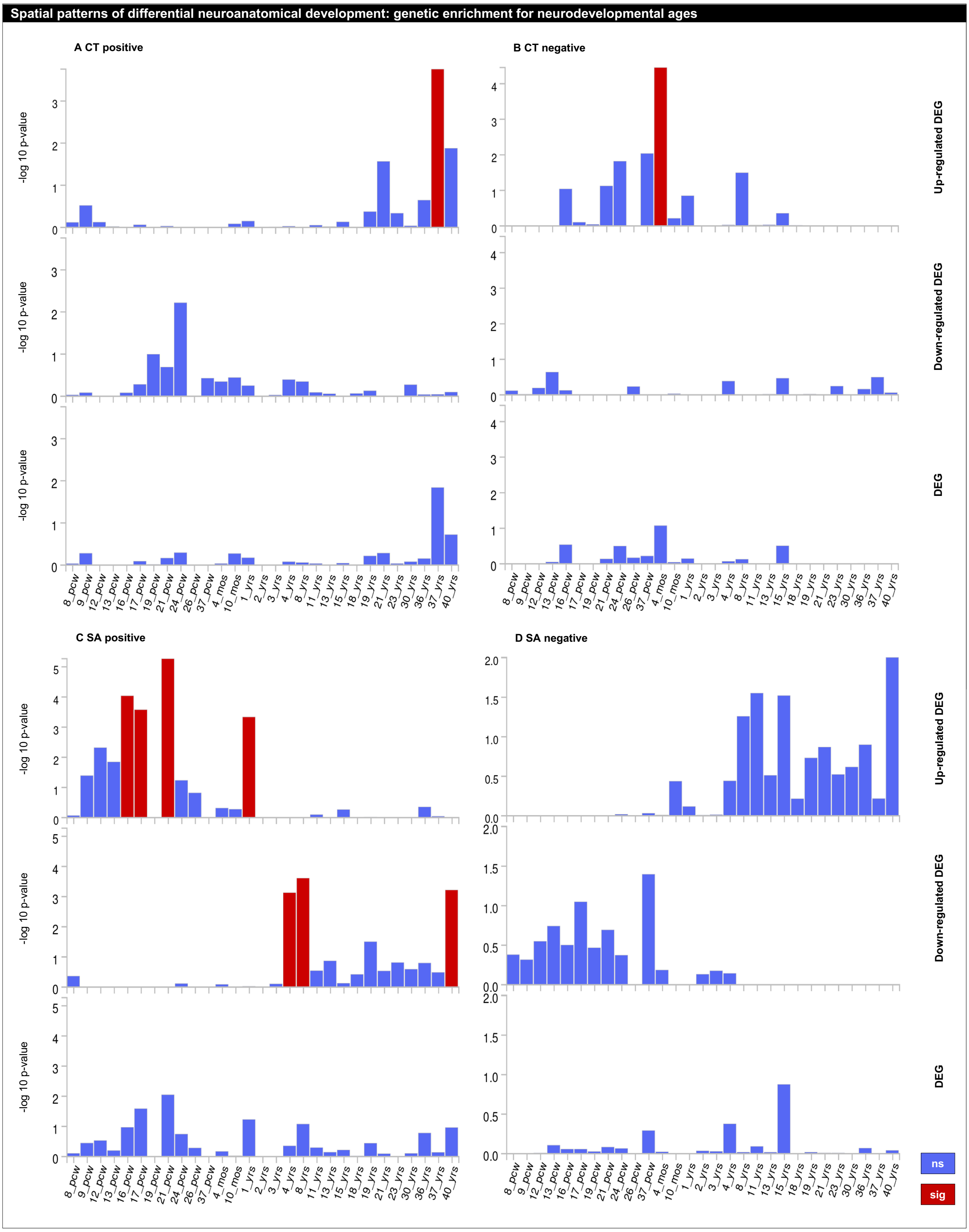

Figure S8 Spatial patterns of neuroanatomical development that differed between groups and their genetic enrichment for neurodevelopmental ages. Association between genes positively and negatively associated with a more neurotypical/less autism-like neuroanatomical pattern in A: cortical thickness (CT), positive association, B: cortical thickness, negative association, C: surface area (SA), positive association, and D: SA, negative association. Each panel shows enrichment for developmental ages. X-axes indicate ages, and y-axes show significance levels (-log 10 p-values). Abbreviations: CT, cortical thickness; DEG, differential gene expression; ns, non-significant; sig, significant.

©2024 Pretzsch CM et al. *JAMA Psychiatry*.

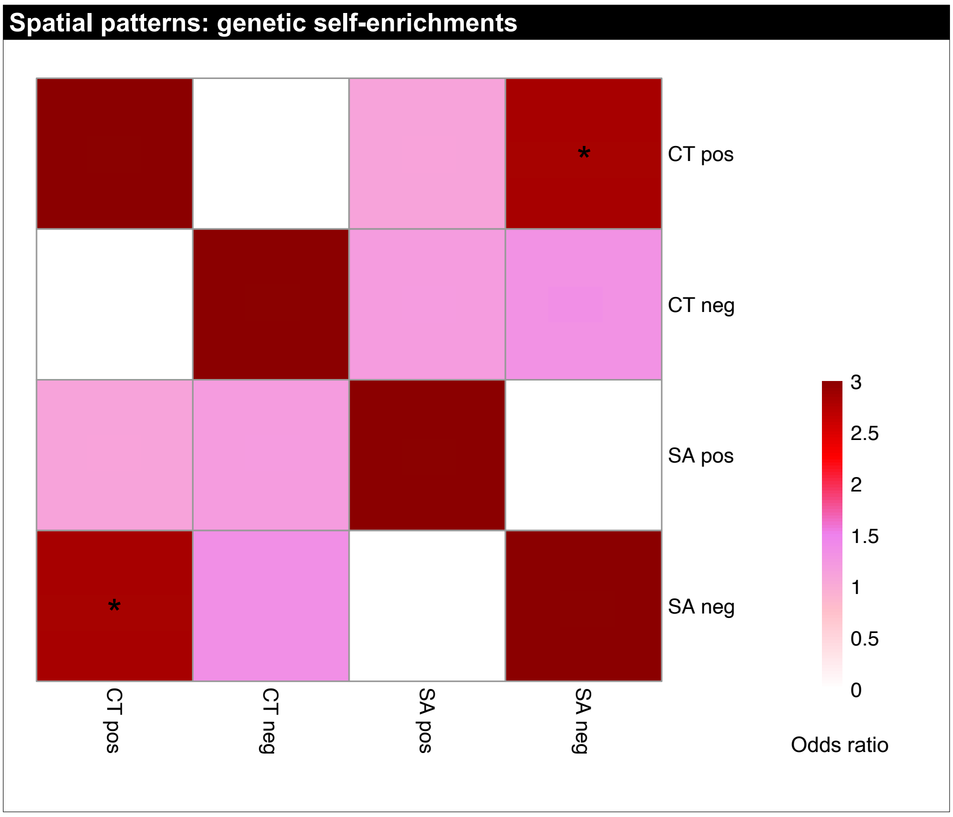

Figure S9 Spatial patterns of neuroanatomical development that differed between groups and their genetic enrichment for each other. Colors indicate enrichment odds ratios, tile symbols indicate false discovery rate (FDR)-corrected p-values. Abbreviations: CT, cortical thickness; neg, negative association with reference group, i.e., a less neurotypical/more autism-like pattern; pos, positive association with reference group, i.e., a more neurotypical/less autism-like pattern; SA, surface area. *p≤0.01

©2024 Pretzsch CM et al. *JAMA Psychiatry*.

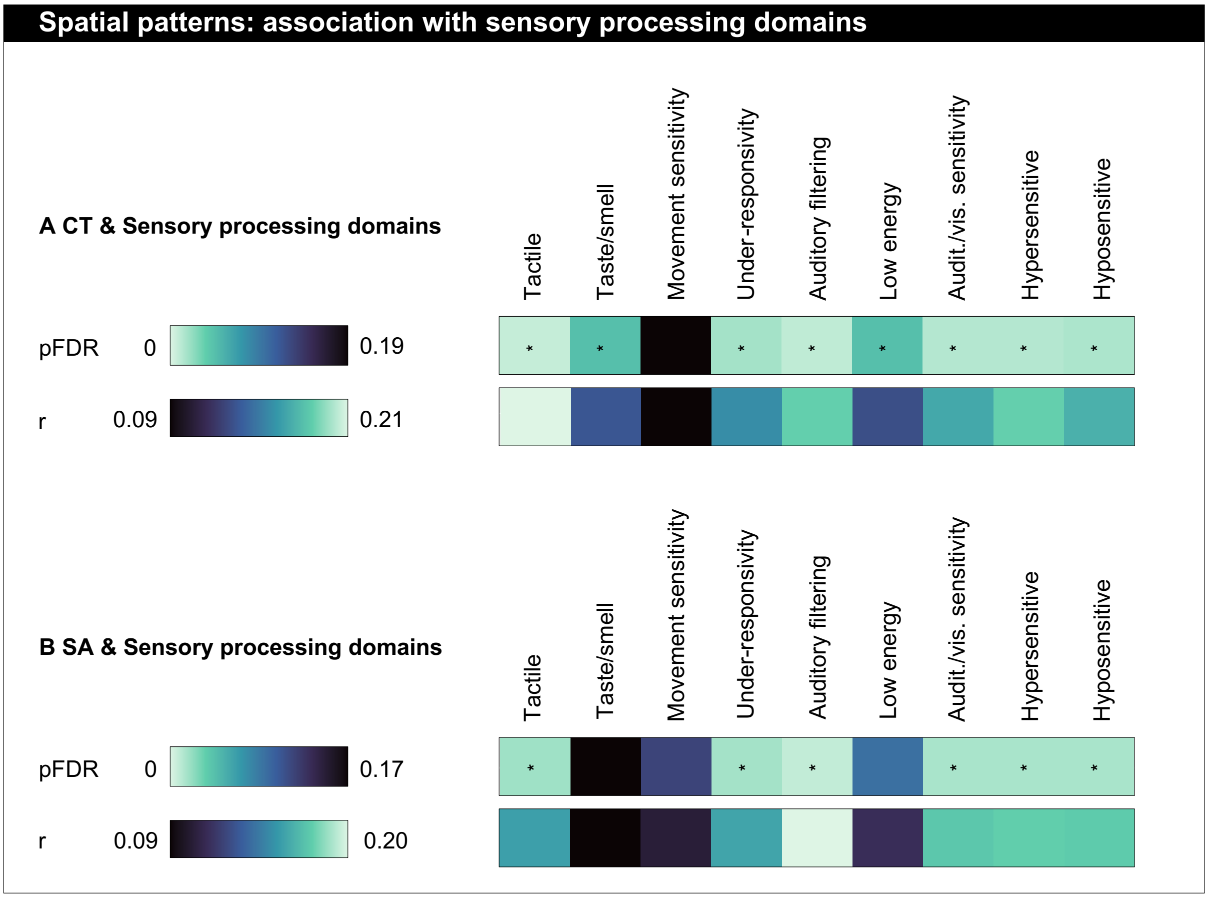

Figure S10 Spatial patterns of neuroanatomical development that differed between groups and their association with sensory processing domains. Associations between A: cortical thickness and sensory processing domains, and B: surface area and sensory processing domains. Colorbars indicate significance (corrected p-values, pFDR) and strength (Pearson's correlation coefficient, r) of correlation. Abbreviations: CT, cortical thickness; pFDR, p-values corrected for false discovery rate; SA, surface area.

©2024 Pretzsch CM et al. *JAMA Psychiatry*.

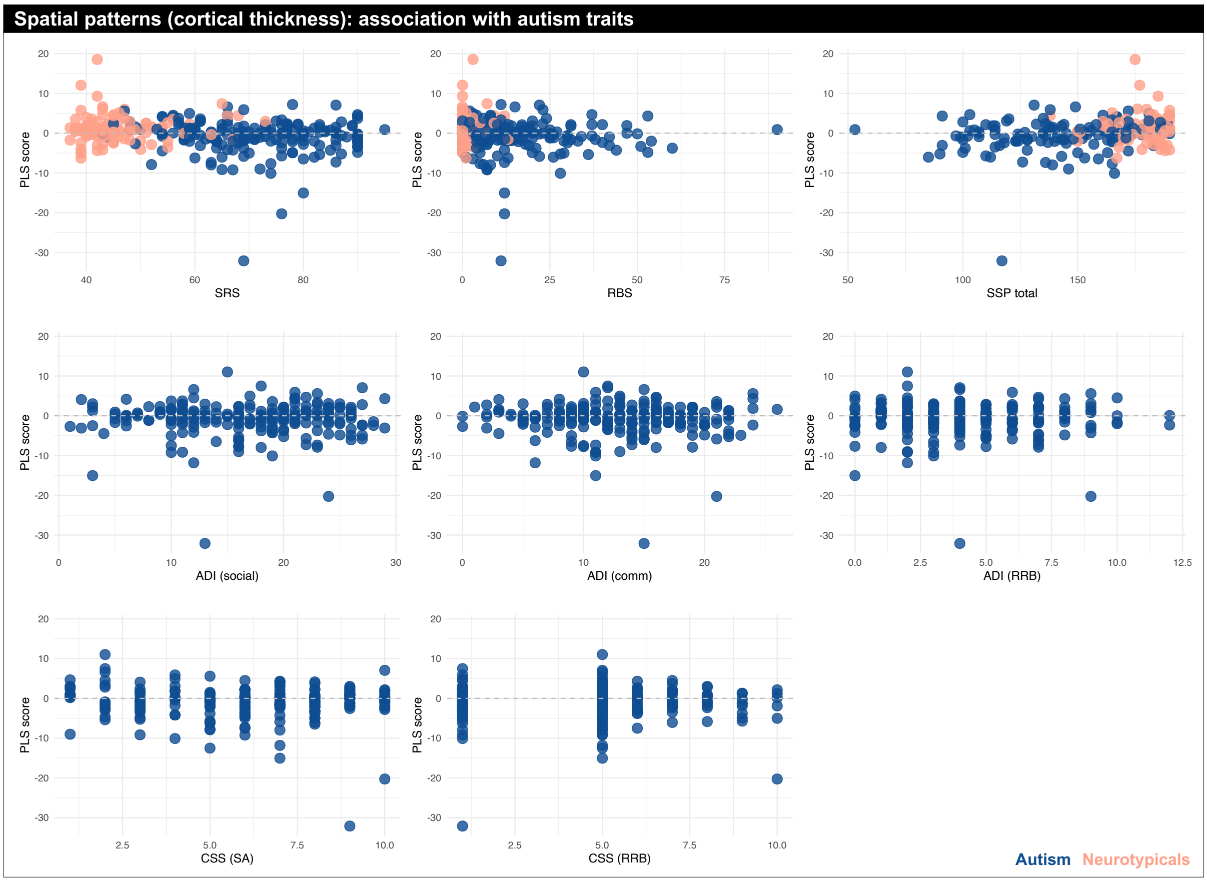

Figure S11 Spatial patterns of cortical thickness development that differed between groups (partial least squares scores) and their association with autism traits. ADI, autism diagnostic interview (comm: communication; RRB: restricted and repetitive behaviours; social: social domain); CSS, autism diagnostic observation schedule calibrated severity score (SA: social affect; rrb: restricted and repetitive behaviours); PLS, partial least squares; RBS, repetitive behaviours scale; SRS, social responsiveness scale; SSP, short sensory profile.

©2024 Pretzsch CM et al. *JAMA Psychiatry*.

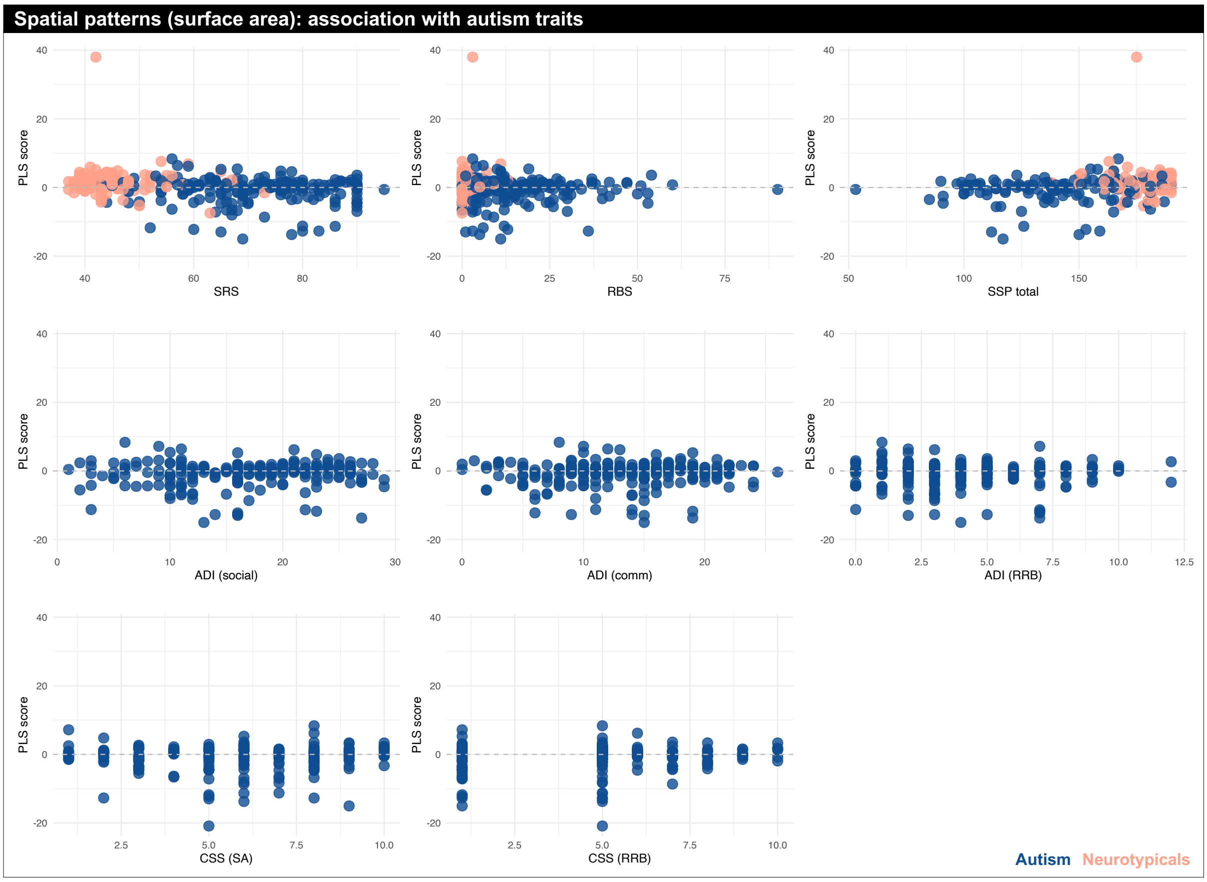

Figure S12 Spatial patterns of surface area development that differed between groups (partial least squares scores) and their association with autism traits. ADI, autism diagnostic interview (comm: communication; RRB: restricted and repetitive behaviours; social: social domain); CSS, autism diagnostic observation schedule calibrated severity score (SA: social affect; rrb: restricted and repetitive behaviours); PLS, partial least squares; RBS, repetitive behaviours scale; SRS, social responsiveness scale; SSP, short sensory profile.

©2024 Pretzsch CM et al. *JAMA Psychiatry*.

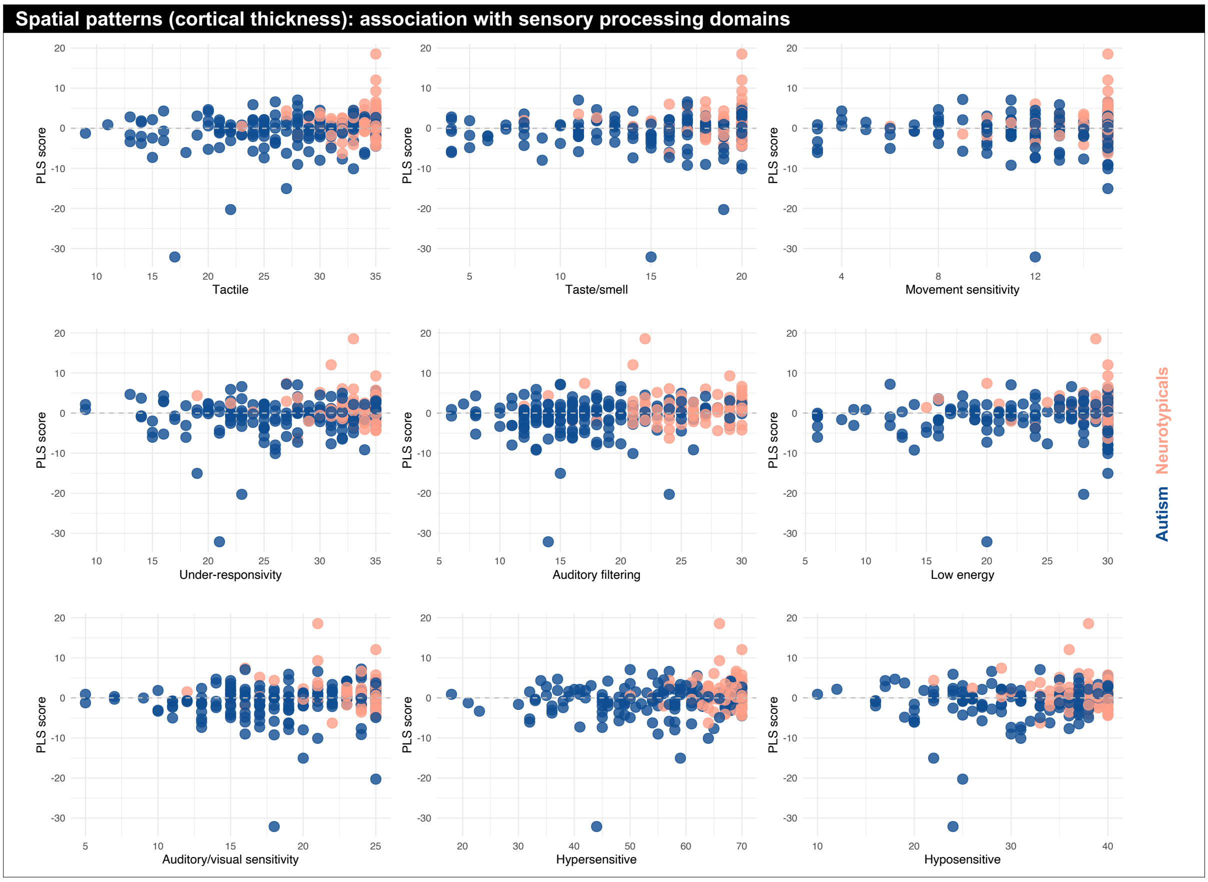

Figure S13 Spatial patterns of cortical thickness development that differed between groups (partial least squares scores) and their association with sensory processing domains. Abbreviations: PLS, partial least squares.

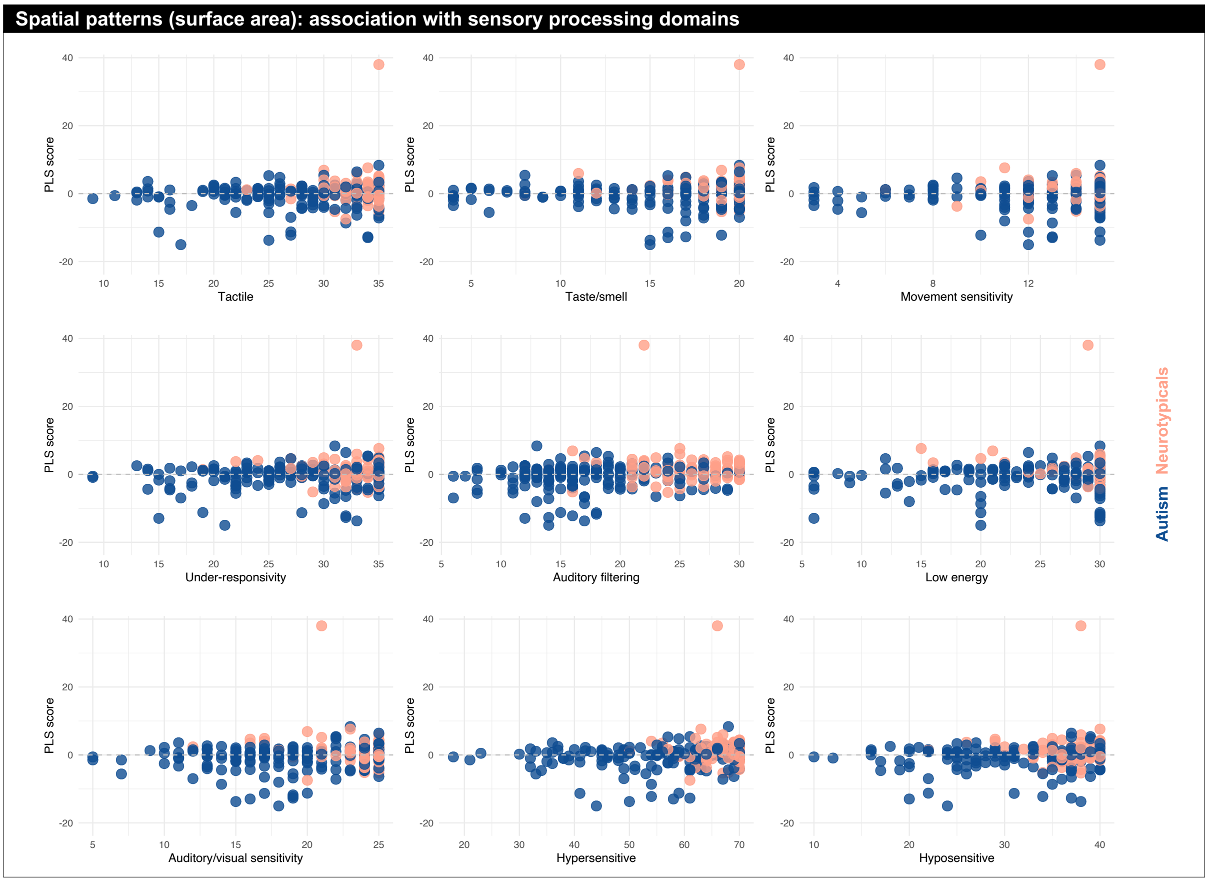

Figure S14 Spatial patterns of surface area development that differed between groups (partial least squares scores) and their association with sensory processing domains. Abbreviations: PLS, partial least squares.

©2024 Pretzsch CM et al. *JAMA Psychiatry*.

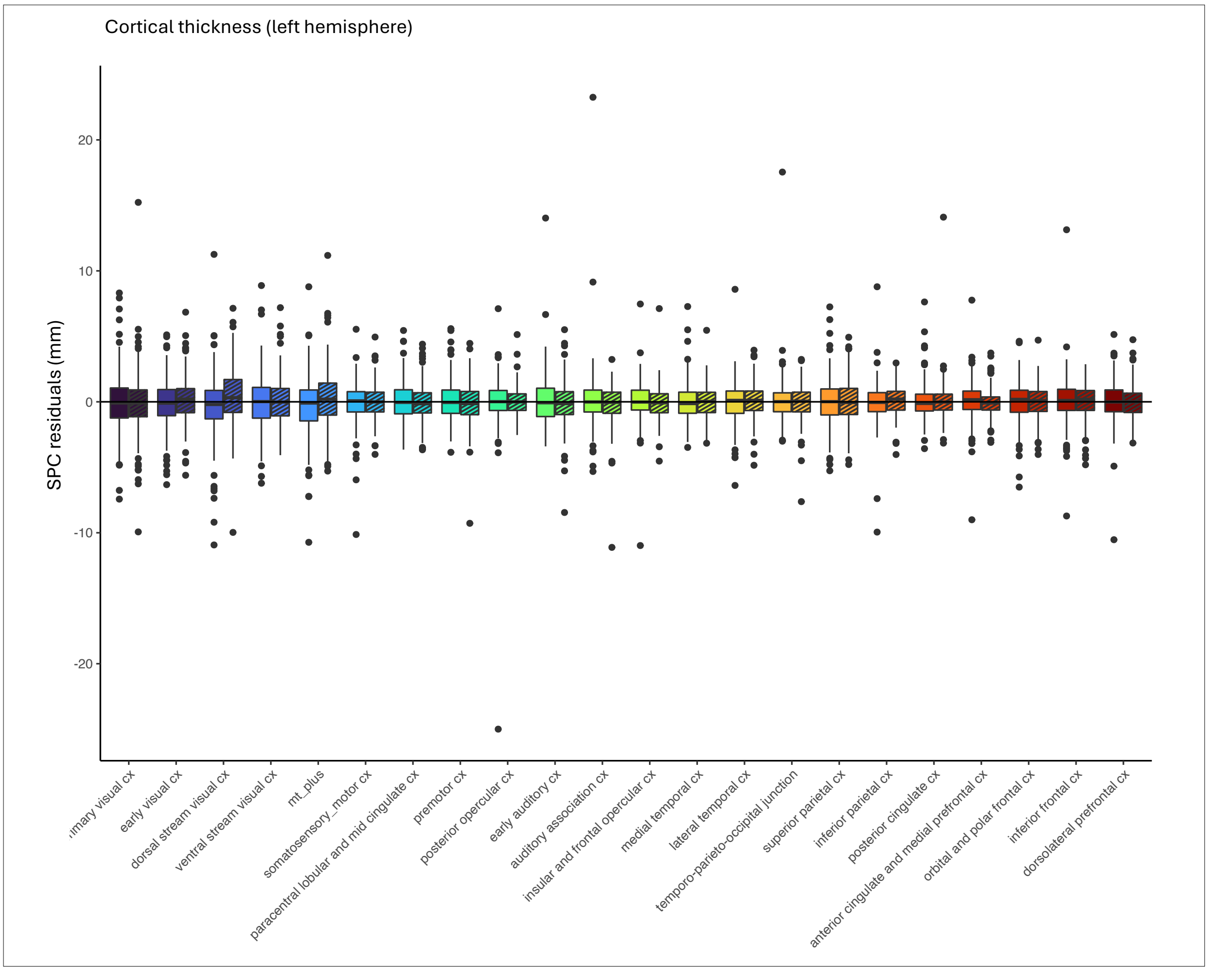

Figure S15 Cortical thickness maturation in the left hemisphere over time (residuals). Y axis depicts the symmetrized percentage change in mm corrected for confounding variables. X axis depicts 22 Glasser areas per hemisphere. Abbreviations: cx, cortex.

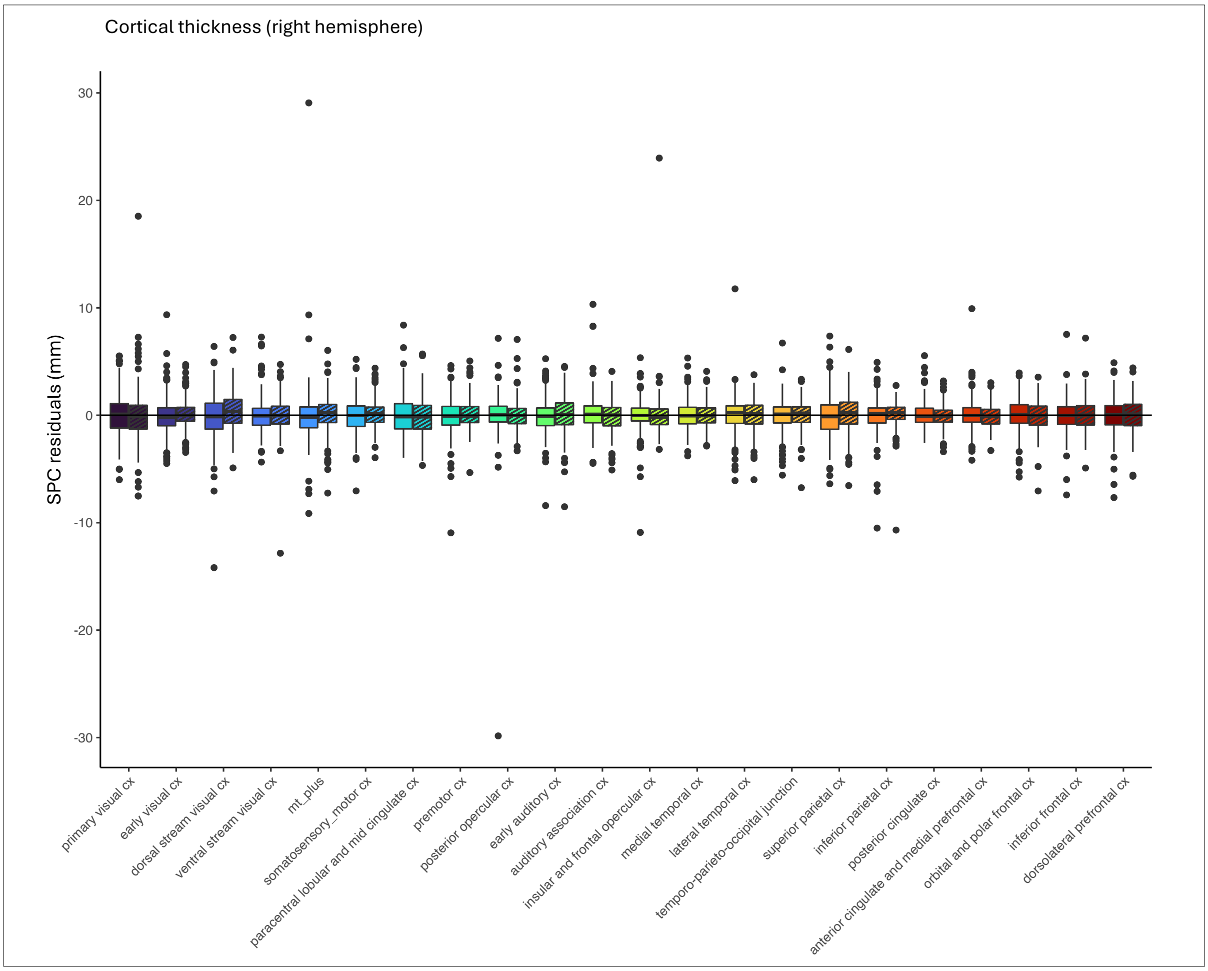

Figure S16 Cortical thickness maturation in the right hemisphere over time (residuals). Y axis depicts the symmetrized percentage change in mm corrected for confounding variables. X axis depicts 22 Glasser areas per hemisphere. Abbreviations: cx, cortex.

©2024 Pretzsch CM et al. *JAMA Psychiatry*.

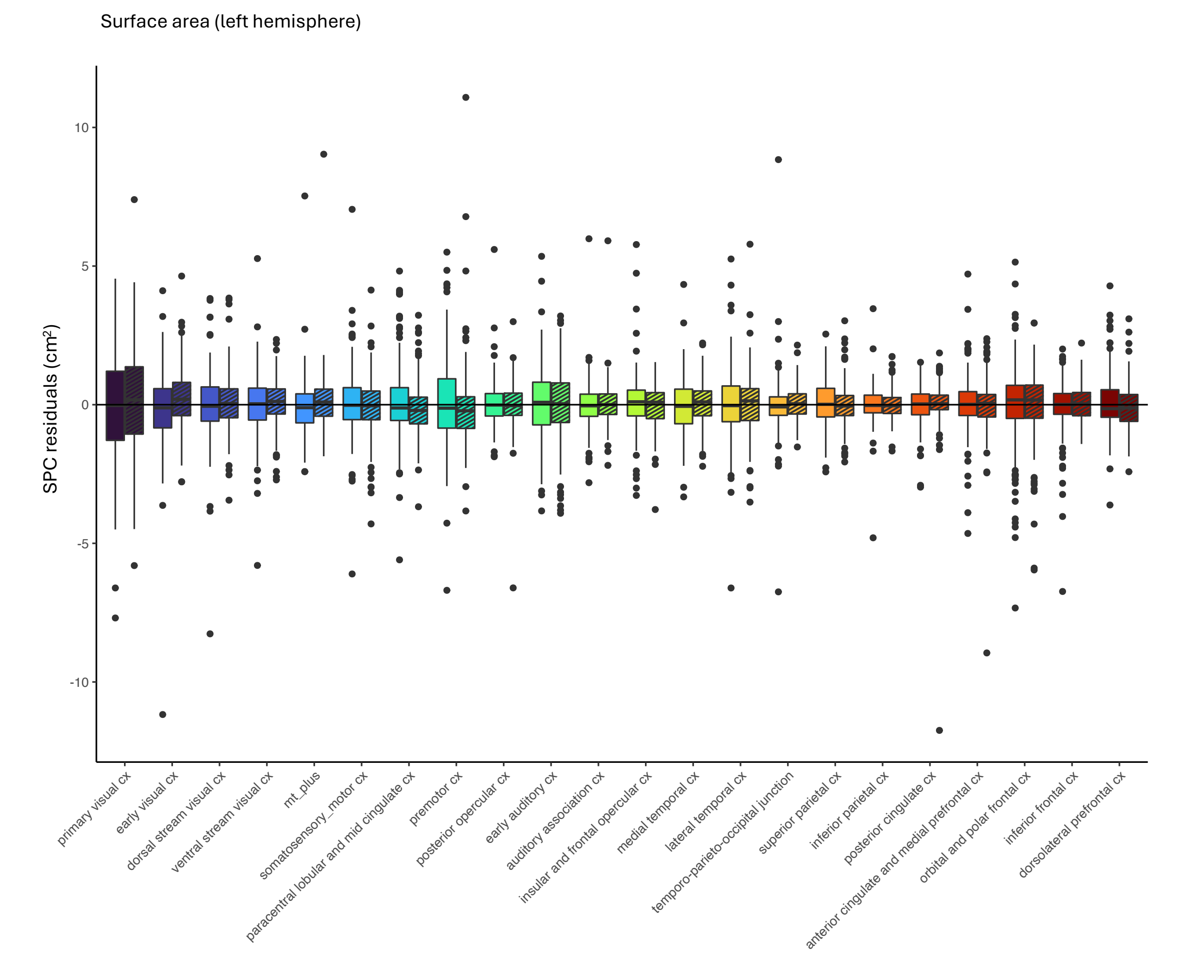

Figure S17 Surface area maturation in the left hemisphere over time (residuals). Y axis depicts the symmetrized percentage change in cm² corrected for confounding variables. X axis depicts 22 Glasser areas per hemisphere. Abbreviations: cx, cortex.

©2024 Pretzsch CM et al. *JAMA Psychiatry*.

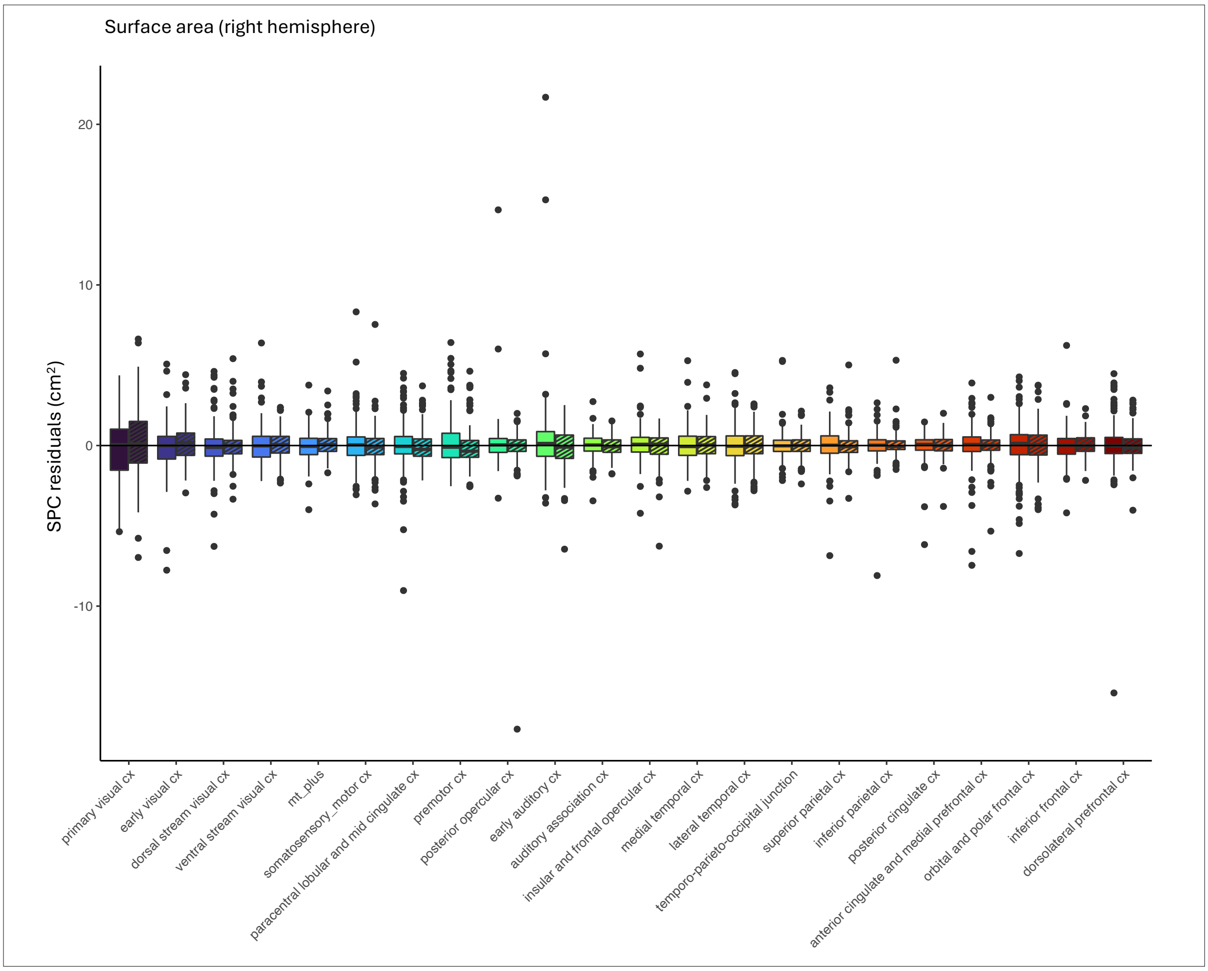

Figure S18 Surface area maturation in the right hemisphere over time (residuals). Y axis depicts the symmetrized percentage change in cm^2 corrected for confounding variables. X axis depicts 22 Glasser areas per hemisphere. Abbreviations: cx, cortex.

©2024 Pretzsch CM et al. *JAMA Psychiatry*.

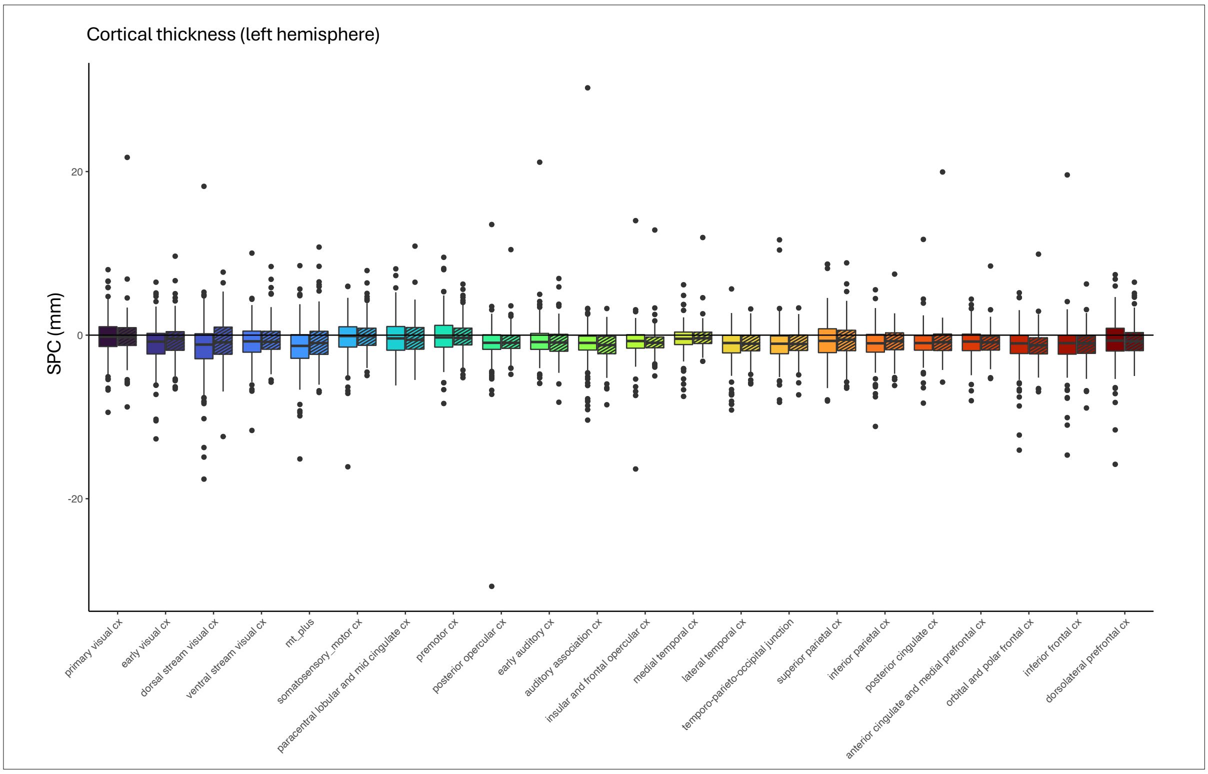

Figure S19 Cortical thickness maturation in the left hemisphere over time. Y axis depicts the symmetrized percentage change in mm. X axis depicts 22 Glasser areas per hemisphere. Abbreviations: cx, cortex.

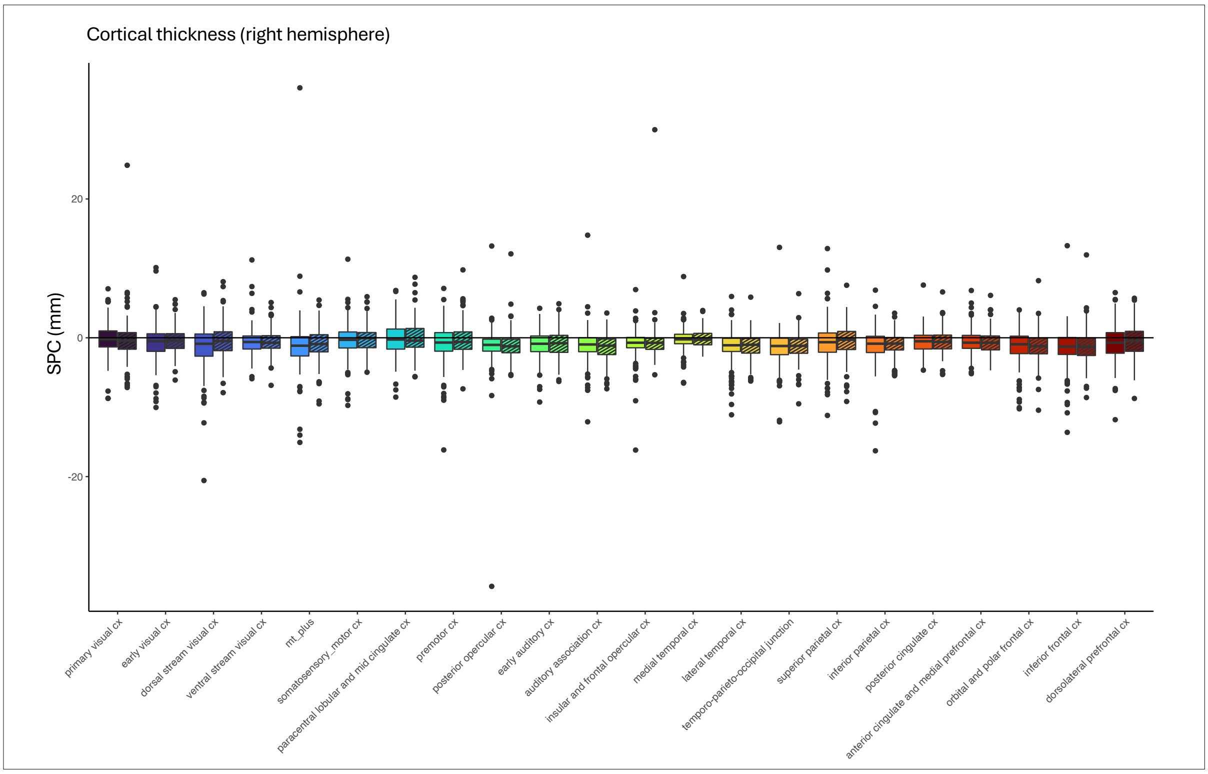

Figure S20 Cortical thickness maturation in the right hemisphere over time. Y axis depicts the symmetrized percentage change in mm. X axis depicts 22 Glasser areas per hemisphere. Abbreviations: cx, cortex.

©2024 Pretzsch CM et al. *JAMA Psychiatry*.

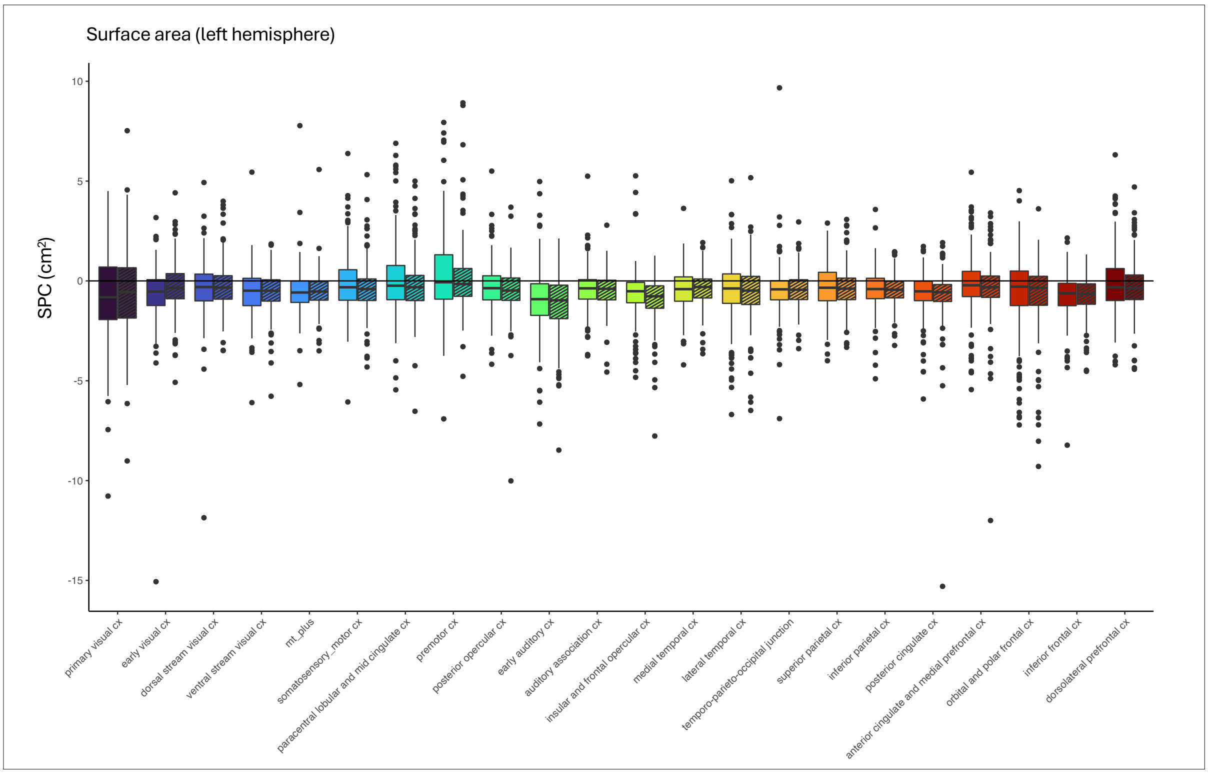

Figure S21 Surface area maturation in the left hemisphere over time. Y axis depicts the symmetrized percentage change in cm^2. X axis depicts 22 Glasser areas per hemisphere. Abbreviations: cx, cortex.

©2024 Pretzsch CM et al. *JAMA Psychiatry*.

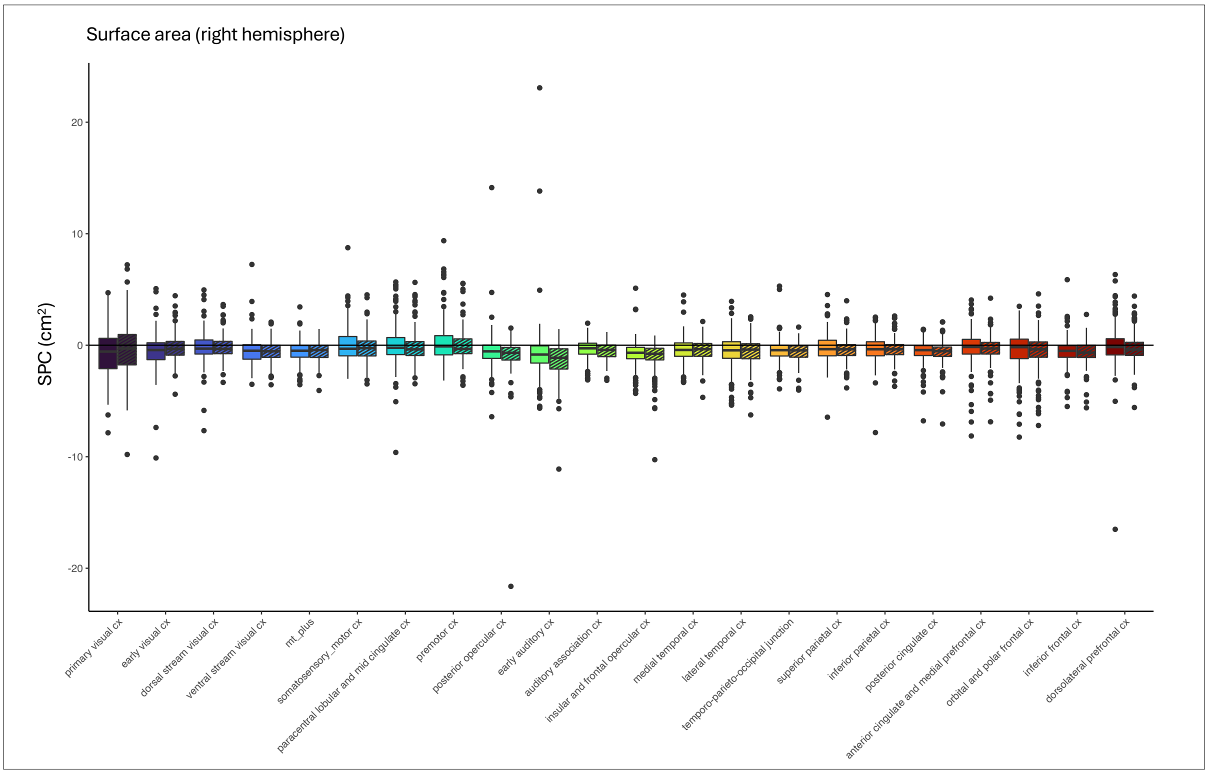

Figure S22 Surface area maturation in the right hemisphere over time. Y axis depicts the symmetrized percentage change in cm^2. X axis depicts 22 Glasser areas per hemisphere. Abbreviations: cx, cortex.

©2024 Pretzsch CM et al. *JAMA Psychiatry*.

eTables

Table S1 Medication

Diagnostic group	Unknown % (n)	No % (n)	Yes % (n)	Medication and Categories
Autism (n = 214)	31% (67)	29% (62)	40% (85)<br><br>One: n=81<br>Two: n=25<br>Three: n=3<br>Missing: n=10	Antidepressant (n=24)<br>• 19 Selective serotonin reuptake inhibitor (SSRI)<br>• 2 Tetracyclic antidepressant (TeCA)<br>• 3 Tricyclic Antidepressant (TCA)<br><br>Antiepileptics (n=6; no additional information)<br><br>Antimigraine preparations (n=3; no additional information)<br><br>Antipsychotics (n=16)<br>• 4 Aripiprazole<br>• 1 Clozapine<br>• 1 Pipamperone<br>• 9 Risperidone<br>• 1 Quetiapine<br><br>Anxiolytics (n=1; no additional information)<br><br>Hypnotics and sedatives (n=28)<br>• 28 Melatonin<br><br>Other analgesics and antipyretics (n=4)<br>• 4 Other analgesics and antipyretics<br><br>Psychostimulants and other drugs used to treat ADHD (n=26)<br>• 2 Atomoxtine<br>• 1 Dexamfetamine<br>• 23 Methylphenidate hydrochloride
Neurotypical (n = 172)	56% (97)	37% (63)	7% (12)<br><br>One: n=9<br>Two: n=1<br>Three: n=1<br>Missing: n=1	Antidepressant (n=3)<br>• 3 Selective serotonin reuptake inhibitor (SSRI)<br><br>Antiepileptics (n=2; no additional information)<br><br>Anxiolytics (n=1; no additional information)<br><br>Drugs used in addictive disorder (n=1; no additional information)<br><br>Hypnotics and sedatives (n=1)<br>• 1 Melatonin<br><br>Other analgesics and antipyretics (n=2)<br><br>Psychostimulants and other drugs used to treat ADHD (n=4)<br>• 1 Atomoxetine<br>• 3 Methylphenidate hydrochloride

Table S2 Spatial components within individual age-groups

Feature	Age group	Component	Correlation with results obtained across age-groups	
			r	p_{FDR}
Cortical thickness	Children	1	0.65	<.001
		2	-0.11	.06
		3	-0.08	.15
		4	-0.21	<.001
		5	-0.08	.15
		6	0.37	<.001
	Adolescents	1	0.76	<.001
		2	-0.26	<.001
	Adults	1	0.38	<.001
		2	0.06	.28
		3	-0.56	<.001
Surface area	Children	1	0.61	<.001
		2	-0.41	<.001
		3	0.29	<.001
		4	-0.54	<.001
		5	0.37	<.001
	Adolescents	1	0.81	<.001
	Adults	1	0.27	<.001
		2	-0.26	<.001
		3	0.54	<.001

©2024 Pretzsch CM et al. *JAMA Psychiatry*.

Table S3 Genes positively associated with a neurotypical maturational pattern: enrichment for cell-types and genes differentially expressed in autism. Abbreviations: CT, cortical thickness; SA, surface area.

Feature	Gene sets		# Genes in Overlap (k)	# Genes in Gene Set (K)	Odds ratio	p-value
CT	Genes	Source				
	Cell-types	Zhong_astrocyte_genelist	5	326	0.9	0.66
		Zhong_microglial_genelist	9	438	1.2	0.33
		Zhong_excitatoryneurons_genelist	0	18	0	1
		Zhong_interneurons_genelist	0	18	0	1
		Zhong_neuroprogenitorcells_genelist	3	142	1.2	0.44
		Zhong_olygodendrocytes_ precursorcells_genelist	2	91	1.3	0.46
		Zhongmicroglialtype1_genelist	6	257	1.4	0.28
		Zhongmicroglialtype2_genelist	7	488	0.8	0.73
		Manno_midbrain_basalcells_genelist	1	79	0.7	0.74
		Manno_midabrain_dopamineneurons_cells	11	506	1.3	0.25
		Manno_midbrain_gabaneurons_genelist	25	1106	1.4	0.09
		Manno_midbrain_5HTneurons_genelist	3	450	0.4	0.98
		Manno_midbrain_radialglial_genelists	8	364	1.3	0.28
		Manno_midbrain_dopamineneurons1_genelist	10	584	1	0.54
		Manno_midbrain_dopamineneurons2_genelist	11	513	1.3	0.26
		Manno_midbrain_radialglial2_genelists	7	325	1.3	0.32
		Manno_midbrain_radialglial3_genelists	14	574	1.5	0.12
	Dys-regulation	ASD_downregulatedgenes	18	764	1.4	0.1
		ASD_upregulated	12	1111	0.6	0.97
SA	Cell-types	Zhong_astrocyte_genelist	4	326	0.6	0.88
		Zhong_microglial_genelist	1	438	1	0.10
		Zhong_excitatoryneurons_genelist	0	18	0	1
		Zhong_interneurons_genelist	0	18	0	1

©2024 Pretzsch CM et al. *JAMA Psychiatry*.

		Zhong_neuroprogenitorcells_genelist	3	142	1.1	0.52
		Zhong_olygodendrocytes_precursorcells_genelist	2	91	1.1	0.53
		Zhong_microglialtype1_genelist	3	257	0.6	0.88
		**Zhong_microglialtype2_genelist**	**18**	**488**	**2**	**0.0074**
		Manno_midbrain_basalcells_genelist	0	79	0	1
		Manno_midabrain_dopamineneurons_cells	10	506	1	0.53
		**Manno_midbrain_gabaneurons_genelist**	**43**	**1106**	**2.2**	**1.1.e-05**
		Manno_midbrain_5HTneurons_genelist	16	450	1.9	0.01
		Manno_midbrain_radialglial_genelists	5	364	0.7	0.84
		**Manno_midbrain_dopamineneurons1_genelist**	**21**	**584**	**1.9**	**0.0053**
		Manno_midbrain_dopamineneurons2_genelist	11	513	1.1	0.41
		Manno_midbrain_radialglial2_genelists	5	325	0.8	0.76
		Manno_midbrain_radialglial3_genelists	10	574	0.9	0.68
	Dys-regulation	ASD_downregulatedgenes	9	764	0.6	0.96
		ASD_upregulated	25	1111	1.2	0.25

©2024 Pretzsch CM et al. *JAMA Psychiatry*.

Table S4 Genes negatively associated with a neurotypical maturational pattern: enrichment for cell-types and genes differentially expressed in autism Abbreviations: CT, cortical thickness; SA, surface area.

Contrast	Gene sets		# Genes in Overlap (k)	# Genes in Gene Set (K)	Odds ratio	p-value
**CT**	**Genes**	**Source**				
	Cell-types	Zhong_astrocyte_genelist	14	326	1.4	0.16
		Zhong_microglial_genelist	42	438	3.4	0.17
		Zhong_excitatoryneurons_genelist	1	18	1.8	0.44
		Zhong_interneurons_genelist	1	18	1.8	0.44
		Zhong_neuroprogenitorcells_genelist	3	142	0.7	0.83
		Zhong	6	91	2.2	0.06
		Zhongmicroglialtype1_genelist	2	257	0.2	1
		**Zhongmicroglialtype2_genelist**	**27**	**488**	**1.8**	**0.0038**
		Manno_midbrain_basalcells_genelist	1	79	0.4	0.92
		**Manno_midabrain_dopamineneurons_cells**	**34**	**506**	**2.3**	**3.30E-05**
		**Manno_midbrain_gabaneurons_genelist**	**46**	**1106**	**1.4**	**0.03**
		**Manno_midbrain_5HTneurons_genelist**	**27**	**450**	**2**	**0.0012**
		Manno_midbrain_radialglial_genelists	14	364	1.2	0.27
		**Manno_midbrain_dopamineneurons1_genelist**	**36**	**584**	**2.1**	**1.20E-04**
		**Manno_midbrain_dopamineneurons2_genelist**	**39**	**513**	**2.6**	**4.40E-07**
		Manno_midbrain_radialglial2_genelists	10	325	1	0.58
		Manno_midbrain_radialglial3_genelists	21	574	1.2	0.28
	Dys-regulation	ASD_downregulatedgenes	8	764	0.3	1
		ASD_upregulated	45	1111	1.3	0.05
**SA**	Cell-types	Zhong_astrocyte_genelist	5	326	1	0.58
		Zhong_microglial_genelist	5	438	0.7	0.82
		Zhong_excitatoryneurons_genelist	0	18	0	1
		Zhong_interneurons_genelist	0	18	0	1

©2024 Pretzsch CM et al. *JAMA Psychiatry*.

		Zhong_neuroprogenitorcells_genelist	4	142	1.8	0.18
		Zhong_olygodendrocytes_precursorcells_genelist	4	91	2.9	0.05
		Zhongmicroglialtype1_genelist	3	257	0.7	0.77
		Zhongmicroglialtype2_genelist	8	488	1	0.50
		Manno_midbrain_basalcells_genelist	1	79	0.8	0.71
		Manno_midabrain_dopamineneurons_cells	8	506	1	0.54
		Manno_midbrain_gabaneurons_genelist	15	1106	0.9	0.75
		Manno_midbrain_5HTneurons_genelist	11	450	1.6	0.09
		Manno_midbrain_radialglial_genelists	4	364	0.7	0.82
		Manno_midbrain_dopamineneurons1_genelist	9	584	1	0.57
		Manno_midbrain_dopamineneurons2_genelist	7	513	0.9	0.7
		Manno_midbrain_radialglial2_genelists	5	325	1	0.58
		Manno_midbrain_radialglial3_genelists	9	574	1	0.55
	Dys-regulation	**ASD_downregulatedgenes**	**38**	**764**	**3.7**	**2.60E-10**
		ASD_upregulated	8	1111	0.4	1

©2024 Pretzsch CM et al. *JAMA Psychiatry*.

Table S5 Genes positively and negatively associated with a neurotypical maturational pattern: enrichment for Gene Ontology (GO) biological processes. Abbreviations: CT, cortical thickness; SA, surface area.

Contrast	Gene Set Name	# Genes in Overlap (k)	# Genes in Gene Set (K)	k/K	p-value	FDR q-value
CT: positive	GOBP_CELLULAR_RESPONSE_TO_STRESS	39	1973	0.0198	2.91E-09	0.0000226
	GOBP_CELL_PROJECTION_ORGANIZATION	30	1576	0.019	0.000000497	0.000918
	GOBP_POSITIVE_REGULATION_OF_RNA_METABOLIC_PROCESS	33	1853	0.0178	0.00000057	0.000918
	GOBP_DNA_REPAIR	17	584	0.0291	0.000000706	0.000918
	GOBP_CHROMATIN_ORGANIZATION	18	652	0.0276	0.000000709	0.000918
	GOBP_CELLULAR_RESPONSE_TO_DNA_DAMAGE_STIMULUS	21	868	0.0242	0.00000071	0.000918
	GOBP_CELL_CYCLE	32	1847	0.0173	0.00000154	0.0017
	GOBP_GROWTH	21	922	0.0228	0.00000184	0.00178
	GOBP_PROTEIN_CONTAINING_COMPLEX_ORGANIZATION	31	1828	0.017	0.00000349	0.00278
	GOBP_DNA_METABOLIC_PROCESS	22	1043	0.0211	0.00000359	0.00278
SA: positive	GOBP_CELL_PROJECTION_ORGANIZATION	44	1576	0.0279	1.88E-13	1.46E-09
	GOBP_GENERATION_OF_NEURONS	40	1443	0.0277	3.25E-12	1.26E-08
	GOBP_NEURON_DEVELOPMENT	33	1108	0.0298	4.68E-11	0.000000121
	GOBP_REGULATION_OF_CELL_PROJECTION_ORGANIZATION	25	684	0.0365	1.87E-10	0.000000362
	GOBP_NEUROGENESIS	40	1667	0.024	2.45E-10	0.00000038
	GOBP_REGULATION_OF_NEURON_PROJECTION_DEVELOPMENT	20	452	0.0442	5.03E-10	0.00000065
	GOBP_CELL_JUNCTION_ORGANIZATION	25	741	0.0337	9.64E-10	0.00000107
	GOBP_CELL_MORPHOGENESIS	27	931	0.029	5.15E-09	0.00000499
	GOBP_POSITIVE_REGULATION_OF_RNA_METABOLIC_PROCESS	39	1853	0.021	1.56E-08	0.0000134
	GOBP_CHROMATIN_ORGANIZATION	21	652	0.0322	4.81E-08	0.0000372
CT: negative	GOBP_REGULATION_OF_TRANSPORT	74	1809	0.0409	6.36E-21	4.93E-17
	GOBP_CELL_CELL_SIGNALING	67	1697	0.0395	3.42E-18	1.33E-14
	GOBP_NEGATIVE_REGULATION_OF_RESPONSE_TO_STIMULUS	61	1714	0.0356	1.38E-14	2.43E-11
	GOBP_CELL_ADHESION	57	1524	0.0374	1.4E-14	2.43E-11
	GOBP_RESPONSE_TO_OXYGEN_CONTAINING_COMPOUND	63	1819	0.0346	1.67E-14	2.43E-11

©2024 Pretzsch CM et al. *JAMA Psychiatry*.

	GOBP_REGULATION_OF_IMMUNE_SYSTEM_PROCESS	56	1493	0.0375	2.16E-14	2.43E-11
	GOBP_SMALL_MOLECULE_METABOLIC_PROCESS	63	1830	0.0344	2.19E-14	2.43E-11
	GOBP_REGULATION_OF_SECRETION	35	635	0.0551	5.17E-14	4.94E-11
	GOBP_MONOATOMIC_ION_TRANSPORT	50	1252	0.0399	5.74E-14	4.94E-11
	GOBP_EXPORT_FROM_CELL	41	905	0.0453	2.22E-13	1.72E-10
SA: negative	GOBP_ESTABLISHMENT_OF_PROTEIN_LOCALIZATION	33	1684	0.0196	7.23E-09	0.000056
	GOBP_REGULATION_OF_CELLULAR_COMPONENT_BIOGENESIS	23	989	0.0233	8.19E-08	0.000317
	GOBP_RESPONSE_TO_OXYGEN_CONTAINING_COMPOUND	31	1819	0.017	0.000000461	0.00119
	GOBP_REGULATION_OF_CELL_JUNCTION_ASSEMBLY	10	214	0.0467	0.0000011	0.00182
	GOBP_REGULATION_OF_TRANSPORT	30	1809	0.0166	0.00000125	0.00182
	GOBP_REGULATION_OF_MONOATOMIC_ION_TRANSPORT	16	599	0.0267	0.00000141	0.00182
	GOBP_CELLULAR_COMPONENT_MORPHOGENESIS	18	767	0.0235	0.00000193	0.00194
	GOBP_REGULATION_OF_MONOATOMIC_CATION_TRANSMEMBRANE_TRANSPORT	12	346	0.0347	0.00000217	0.00194
	GOBP_REGULATION_OF_MONOATOMIC_ION_TRANSMEMBRANE_TRANSPORT	14	478	0.0293	0.00000225	0.00194
	GOBP_INTRACELLULAR_TRANSPORT	27	1591	0.017	0.00000289	0.00224

©2024 Pretzsch CM et al. *JAMA Psychiatry*.

Table S6 Association between maturational patterns and behavioural measures. Abbreviations: ADI, autism diagnostic interview (comm: communication subscale; rrb: restricted and repetitive behaviour subscale; social: social subscale); CSS, autism diagnostic observation schedule calibrated severity score (sa: social affect subscale; rrb: restricted and repetitive behaviour subscale; total: overall score); RBS, repetitive behaviours scale; SRS, social responsiveness scale; SSP, short sensory profile.

Feature	Measure	P-value	Lower CI	Upper CI	r	P-value (FDR-corrected)
Cortical thickness	SRS	0	-0.32	-0.1	-0.22	**.002**
	RBS	.029	-0.25	-0.01	-0.13	.077
	SSP TOTAL	.001	0.09	0.35	0.23	**.004**
	ADI (SOCIAL)	.776	-0.12	0.15	0.02	.929
	ADI (COMM)	.599	-0.17	0.1	-0.04	.929
	ADI (RRB)	.831	-0.15	0.12	-0.01	.929
	CSS (SOCIAL)	.175	-0.24	0.04	-0.1	.350
	CSS (RRB)	.929	-0.14	0.15	0.01	.929
Surface area	SRS	0	-0.35	-0.13	-0.24	**.0003**
	RBS	.076	-0.22	0.01	-0.11	.151
	SSP TOTAL	.002	0.08	0.34	0.22	**.007**
	ADI (SOCIAL)	.294	-0.06	0.21	0.07	.470
	ADI (COMM)	.626	-0.1	0.17	0.03	.834
	ADI (RRB)	.928	-0.13	0.14	0.01	.928
	CSS (SOCIAL)	.753	-0.12	0.16	0.02	.861
	CSS (RRB)	.016	0.03	0.31	0.17	**.043**

©2024 Pretzsch CM et al. *JAMA Psychiatry*.

Table S7 Demographics of the BrainMapASD sample. Data are expressed as mean ± standard deviation (n, unless as specified at the top of the column). Abbreviations: ADI, autism diagnostic interview (comm: communication subscale; rrb: restricted and repetitive behaviour subscale; social: social subscale); CSS, autism diagnostic observation schedule calibrated severity score (total: overall score); CT, cortical thickness; F, female; FSIQ, full-scale intelligence quotient; M, male; RBSR, repetitive behaviours scale-revised; SA, surface area; T1, measure at timepoint 1; T2, measure at timepoint 2; P-values are not corrected for multiple comparisons.

BrainMapASD				
**Measure At T1 unless otherwise specified**	**Autism N = 49**	**Neurotypicals N = 97**	**Test statistic (Autism vs Neurotypicals)**	
ADI comm	13.08 ± 4.3			
ADI RRB	4.98 ± 2.5			
ADI social	16.92 ± 5.0			
Age T1 (yrs)	14.31 ± 2.4	14.10 ± 2.5	F(1)=0.225	p=.636
Age T2 (yrs)	16.53 ± 2.4	16.36 ± 2.7	F(1)=0.140	p=.709
CSS total	5.86 ± 2.4			
FSIQ	105.76 ± 15.8 (1 IQ<70)	110.50 ± 13.3 (1IQ<70)	F(1)=3.630	p=.059
Mean CT (mm)	2.74 ± 0.1	2.73 ± 0.1	F(1)=0.623	p=.431
RBSR total	24.18 ± 18.1 (n=45)	2.73 ± 6.5 (n=90)	F(1)=101.015	p<.001
Sex	7 F, 42 M	39 F, 58 M	X^2(1)=10.136	p<.001
SRS total raw score	91.98 ± 28.7 (n=46)	18.13 ± 13.4 (n=92)	F(1)=427.489	p<.001
T2-T1 (yrs)	2.19 ± 0.5	2.16 ± 0.3	F(1)=0.102	p=.749
Total SA (cm^2)	1825.55 ± 156.7	1859.80 ± 180.2	F(1)=1.207	p=.274

©2024 Pretzsch CM et al. *JAMA Psychiatry*.

Table S8 Demographics of the LEAP AIMS sub-sample matched in age to BrainMapASD. Data are expressed as mean ± standard deviation (n, unless as specified at the top of the column). Abbreviations: ADI, autism diagnostic interview (comm: communication subscale; rrb: restricted and repetitive behaviour subscale; social: social subscale); CSS, autism diagnostic observation schedule calibrated severity score (total: overall score); CT, cortical thickness; F, female; FSIQ, full-scale intelligence quotient; M, male; RBSR, repetitive behaviours scale-revised; SA, surface area; T1, measure at timepoint 1; T2, measure at timepoint 2; P-values are not corrected for multiple comparisons.

LEAP n=185				
**Measure At T1 unless otherwise specified**	**Autism N = 104**	**Neurotypicals N = 81**	**Test statistic (Autism vs Neurotypicals)**	
ADI comm	13.77 ± 5.6 (n=103)			
ADI RRB	4.32 ± 2.6 (n=103)			
ADI social	18.56 ± 6.1 (n=103)			
Age T1 (yrs)	15.21± 2.1	15.21 ± 2.1	F(1)=0.000	p=.997
Age T2 (yrs)	16.81 ± 2.1	16.74 ± 2.2	F(1)=0.047	p=.829
CSS total	5.56 ± 2.7 (n=89)			
FSIQ	99.15 ± 19.6	104.95 ± 16.1	F(1)=4.640	p=.033
Mean CT (mm)	2.71 ± 0.1	2.70n ± 0.1	F(1)=0.239	p=.628
RBSR total	16.73 ± 13.4 (n=91)	1.19 ± 2.4 ((n=63)	F(1)=82.543	p=.001
Sex	22 F, 82 M	30 F, 52 M	X^2(1)=5.685	p=.017
SRS total raw score	95.17 ± 30.3 (n=92)	21.88 ± 17.8 (n=64)	F(1)=302.238	p=.001
T2-T1 (yrs)	1.60 ± 0.3	1.53 ± 0.3	F(1)=2.868	p=.092
Total SA (cm^2)	2326.49 ± 234.11	2330.91 ± 233.1	F(1)=0.016	p=.899

©2024 Pretzsch CM et al. *JAMA Psychiatry*.

Table S9 Comparison of demographics between the original (LEAP) and the replication (BrainMapASD) sample. Data are expressed as mean ± standard deviation (n, unless as specified at the top of the column). Abbreviations: ADI, autism diagnostic interview (comm: communication subscale; rrb: restricted and repetitive behaviour subscale; social: social subscale); CSS, autism diagnostic observation schedule calibrated severity score (total: overall score); CT, cortical thickness; F, female; FSIQ, full-scale intelligence quotient; M, male; RBSR, repetitive behaviours scale-revised; SA, surface area; T1, measure at timepoint 1; T2, measure at timepoint 2; P-values are not corrected for multiple comparisons.

**Original (LEAP) vs Replication (BrainMapASD) sample**				
	**Autism**		**Neurotypicals**	
**Measure At T1 unless otherwise specified**	**Test statistic (LEAP vs BrainMapASD)**		**Test statistic (LEAP vs BrainMapASD)**	
ADI comm	F(1)=0.008	p=.928		
ADI RRB	F(1)=4.388	p=.037		
ADI social	F(1)=0.035	p=.852		
Age T1 (yrs)	F(1)=14.554	p<.001	F(1)=13.504	p<.001
Age T2 (yrs)	F(1)=9.081	p=.003	F(1)=6.412	p=.012
CSS total	F(1)=2.482	p=.117		
FSIQ	F(1)=2.311	p=.130	F(1)=3.095	p=.080
Mean CT (mm)	F(1)=9.628	p<.002	F(1)=9.397	p=.002
RBSR total	F(1)=11.387	p<.001	F(1)=3.140	p=.078
Sex	$X^2(1)$=3.971	p<.046	$X^2(1)$=0.236	p<.627
SRS raw score	F(1)=0.016	p=.901	F(1)=3.542	p=.061
T2-T1 (yrs)	F(1)=129.963	p<.001	F(1)=223.340	p<.001
Total SA (cm^2)	F(1)=175.922	p<.001	F(1)=293.267	p<.001

Table S10 - attached separately due to size.
Tab1: For each participant, for each Glasser region, this includes the average cortical thickness (presented separately for the left and right hemisphere). Resid refers to 'residuals', i.e., values that are corrected for age, sex, site, etc., (please refer to methods for more information regarding the statistical models). Tab2: For each participant, for each Glasser region, this includes the average surface area (presented separately for the left and right hemisphere). Resid refers to 'residuals', i.e., values that are corrected for age, sex, site, etc., (please refer to methods for more information regarding the statistical models). Tab3: Across groups and participants, for each Glasser area, this includes the average loading (presented separately for each feature [cortical thickness and surface area] and each hemisphere [left and right]). Tab4: Across groups and participants, for each Glasser region, this includes the average loading (presented separately for each feature [cortical thickness and surface area] and each hemisphere [left and right]).

©2024 Pretzsch CM et al. *JAMA Psychiatry*.

eReferences

1 American Psychiatric Association. (ed American Psychiatric Association) (American Psychiatric Association,, Washington, DC, 2013).

2 World Health Organization. (ed World Health Organization) (World Health Organization, Geneva, 1992).

3 Simonoff, E. *et al.* Psychiatric disorders in children with autism spectrum disorders: prevalence, comorbidity, and associated factors in a population-derived sample. *J Am Acad Child Adolesc Psychiatry* **47**, 921-929 (2008). https://doi.org:10.1097/CHI.0b013e318179964f

4 Wong, A. Y. *et al.* The variation of psychopharmacological prescription rates for people with autism spectrum disorder (ASD) in 30 countries. *Autism Res* **7**, 543-554 (2014). https://doi.org:10.1002/aur.1391

5 Frazier, T. W. *et al.* Prevalence and correlates of psychotropic medication use in adolescents with an autism spectrum disorder with and without caregiver-reported attention-deficit/hyperactivity disorder. *J Child Adolesc Psychopharmacol* **21**, 571-579 (2011). https://doi.org:10.1089/cap.2011.0057

6 Bieneck, V. *et al.* Longitudinal Changes in Cortical Thickness in Adolescents with Autism Spectrum Disorder and Their Association with Restricted and Repetitive Behaviors. *Genes (Basel)* **12** (2021). https://doi.org:10.3390/genes12122024

7 Reuter, M., Schmansky, N. J., Rosas, H. D. & Fischl, B. Within-subject template estimation for unbiased longitudinal image analysis. *Neuroimage* **61**, 1402-1418 (2012). https://doi.org:10.1016/j.neuroimage.2012.02.084

8 Reuter, M. & Fischl, B. Avoiding asymmetry-induced bias in longitudinal image processing. *Neuroimage* **57**, 19-21 (2011). https://doi.org:10.1016/j.neuroimage.2011.02.076

9 Dale, A. M., Fischl, B. & Sereno, M. I. Cortical surface-based analysis. I. Segmentation and surface reconstruction. *Neuroimage* **9**, 179-194 (1999). https://doi.org:10.1006/nimg.1998.0395

10 Fischl, B. FreeSurfer. *Neuroimage* **62**, 774-781 (2012). https://doi.org:10.1016/j.neuroimage.2012.01.021

11 Fischl, B., Sereno, M. I., Tootell, R. B. & Dale, A. M. High-resolution intersubject averaging and a coordinate system for the cortical surface. *Hum Brain Mapp* **8**, 272-284 (1999).

12 Segonne, F. *et al.* A hybrid approach to the skull stripping problem in MRI. *Neuroimage* **22**, 1060-1075 (2004). https://doi.org:10.1016/j.neuroimage.2004.03.032

13 Winkler, A. M. *et al.* Measuring and comparing brain cortical surface area and other areal quantities. *Neuroimage* **61**, 1428-1443 (2012). https://doi.org:10.1016/j.neuroimage.2012.03.026

14 Berry, D. A. & Ayers, G. D. Symmetrized percent change for treatment comparisons. *The American Statistician* **60**, 27-31 (2006).

15 Liem, F. *et al.* Reliability and statistical power analysis of cortical and subcortical FreeSurfer metrics in a large sample of healthy elderly. *Neuroimage* **108**, 95-109 (2015). https://doi.org:10.1016/j.neuroimage.2014.12.035

16 Johnson, W. E., Li, C. & Rabinovic, A. Adjusting batch effects in microarray expression data using empirical Bayes methods. *Biostatistics* **8**, 118-127 (2007). https://doi.org:10.1093/biostatistics/kxj037

17 Glasser, M. F. *et al.* A multi-modal parcellation of human cerebral cortex. *Nature* **536**, 171-178 (2016). https://doi.org:10.1038/nature18933

18 Van Essen, D. C. & Glasser, M. F. in *Cerebrum: the Dana forum on brain science.* (Dana Foundation).
19 Desikan, R. S. *et al.* An automated labeling system for subdividing the human cerebral cortex on MRI scans into gyral based regions of interest. *Neuroimage* **31**, 968-980 (2006). https://doi.org:10.1016/j.neuroimage.2006.01.021
20 Destrieux, C., Fischl, B., Dale, A. & Halgren, E. Automatic parcellation of human cortical gyri and sulci using standard anatomical nomenclature. *Neuroimage* **53**, 1-15 (2010). https://doi.org:10.1016/j.neuroimage.2010.06.010
21 Tzourio-Mazoyer, N. *et al.* Automated anatomical labeling of activations in SPM using a macroscopic anatomical parcellation of the MNI MRI single-subject brain. *Neuroimage* **15**, 273-289 (2002). https://doi.org:10.1006/nimg.2001.0978
22 Abdi, H. Partial least squares regression and projection on latent structure regression (PLS Regression). *Wiley interdisciplinary reviews: computational statistics* **2**, 97-106 (2010).
23 Mei, T. *et al.* Gray matter covariations and core symptoms of autism: the EU-AIMS Longitudinal European Autism Project. *Mol Autism* **11**, 86 (2020). https://doi.org:10.1186/s13229-020-00389-4
24 Sydnor, V. J. *et al.* Neurodevelopment of the association cortices: Patterns, mechanisms, and implications for psychopathology. *Neuron* **109**, 2820-2846 (2021). https://doi.org:10.1016/j.neuron.2021.06.016
25 Pretzsch, C. M. *et al.* Neurobiological Correlates of Change in Adaptive Behavior in Autism. *Am J Psychiatry* **179**, 336-349 (2022). https://doi.org:10.1176/appi.ajp.21070711
26 Pretzsch, C. M. *et al.* Cross-sectional and longitudinal neuroanatomical profiles of distinct clinical (adaptive) outcomes in autism. *Mol Psychiatry* **28**, 2158-2169 (2023). https://doi.org:10.1038/s41380-023-02016-z
27 Nunes, A. S. *et al.* Atypical age-related changes in cortical thickness in autism spectrum disorder. *Sci Rep* **10**, 11067 (2020). https://doi.org:10.1038/s41598-020-67507-3
28 Postema, M. C. *et al.* Altered structural brain asymmetry in autism spectrum disorder in a study of 54 datasets. *Nat Commun* **10**, 4958 (2019). https://doi.org:10.1038/s41467-019-13005-8
29 Constantino, J. N. & Gruber, C. P. *Social responsiveness scale : SRS-2*. Second edition edn, (Western Psychological Services, 2012).
30 Bodfish, J. W., Symons, F. J., Parker, D. E. & Lewis, M. H. Varieties of repetitive behavior in autism: Comparisons to mental retardation. *Journal of Autism and Developmental Disorders* **30**, 237-243 (2000). https://doi.org:Doi 10.1023/A:1005596502855
31 Dunn, W. *Short sensory profile*. (1999).
32 Lord, C., Rutter, M. & Le Couteur, A. Autism Diagnostic Interview-Revised: a revised version of a diagnostic interview for caregivers of individuals with possible pervasive developmental disorders. *J Autism Dev Disord* **24**, 659-685 (1994).
33 Hawrylycz, M. J. *et al.* An anatomically comprehensive atlas of the adult human brain transcriptome. *Nature* **489**, 391-399 (2012). https://doi.org:10.1038/nature11405
34 Gorgolewski, K. "Tight fitting genes: finding relations between statistical maps and gene expression patterns" presented at the 20th Annual Meeting of the Organization for Human Brain Mapping, Hamburg, Germany, 8-12 June 2014. (2014).
35 Zhang, Y. *et al.* An RNA-sequencing transcriptome and splicing database of glia, neurons, and vascular cells of the cerebral cortex. *J Neurosci* **34**, 11929-11947 (2014). https://doi.org:10.1523/JNEUROSCI.1860-14.2014
36 La Manno, G. *et al.* Molecular Diversity of Midbrain Development in Mouse, Human, and Stem Cells. *Cell* **167**, 566-580 e519 (2016). https://doi.org:10.1016/j.cell.2016.09.027

©2024 Pretzsch CM et al. *JAMA Psychiatry*.

37 Gandal, M. J. *et al*. Transcriptome-wide isoform-level dysregulation in ASD, schizophrenia, and bipolar disorder. *Science* **362** (2018). https://doi.org:10.1126/science.aat8127
38 Lord, C. *et al*. The autism diagnostic observation schedule-generic: a standard measure of social and communication deficits associated with the spectrum of autism. *J Autism Dev Disord* **30**, 205-223 (2000).
39 Lord, C. *et al*. Autism diagnostic observation schedule. *Journal of Autism and Developmental Disorders* (2012).
40 Bishop, S. L. & Lord, C. Commentary: Best practices and processes for assessment of autism spectrum disorder - the intended role of standardized diagnostic instruments. *J Child Psychol Psychiatry* **64**, 834-838 (2023). https://doi.org:10.1111/jcpp.13802

©2024 Pretzsch CM et al. *JAMA Psychiatry*.
