# Supplementary material for: Patterns of Brain Maturation in Autism and Their Molecular Associations
Source: JAMA Psychiatry. 2024 Oct 16;81(12):1253–64. doi: 10.1001/jamapsychiatry.2024.3194 (PMC11581727; doi:10.1001/jamapsychiatry.2024.3194)
Supplement: Supplement 3. — Nonauthor Collaborators. EU-AIMS LEAP Group. [file jamapsychiatry-e243194-s003.pdf]

Supplemental Online Content: Nonauthor Collaborators
*First name, last name, and suffix (if applicable) are required and will appear in PubMed.

***Group Name(s): EU-AIMS LEAP Group**

| ***First Name and Middle Initial(s)** | ***Last Name** | ***Suffix (eg, Jr, III)** | Academic Degrees | Institution | Location (city, state/province, country) | Role or Contribution, eg, chair, principal investigator | Group (if more than 1 Group listed in the byline) and/or Subgroup (eg, Steering Committee) |
|---|---|---|---|---|---|---|---|
| Jumana | Ahmad | | Dr | University of Greenwich | London, UK | Co-investigator | EU-AIMS LEAP |
| Sara | Ambrosino | | Dr | University Medical Center Utrecht | Utrecht, Netherlands | Co-investigator | EU-AIMS LEAP |
| Bonnie | Auyeung | | Dr | University of Edinburgh | Edinburgh, UK | Co-investigator | EU-AIMS LEAP |
| Tobias | Banaschewski | | Dr | University of Mannheim | Mannheim, Germany | Co-investigator | EU-AIMS LEAP |
| Simon | Baron-Cohen | | Dr | University of Cambridge | Cambridge, UK | Co-investigator | EU-AIMS LEAP |
| Sarah | Baumeister | | Dr | University of Mannheim | Mannheim, Germany | Co-investigator | EU-AIMS LEAP |
| Christian F. | Beckmann | | Dr | Radboud University | Nijmegen, Netherlands | Co-investigator | EU-AIMS LEAP |
| Sven | Bölte | | Dr | Karolinska Institute | Stockholm, Sweden | Co-investigator | EU-AIMS LEAP |
| Thomas | Bourgeron | | Dr | Institut Pasteur | Paris, France | Co-investigator | EU-AIMS LEAP |
| Carsten | Bours | | Dr | Radboud University | Nijmegen, Netherlands | Co-investigator | EU-AIMS LEAP |
| Michael | Brammer | | Dr | King's College London | London, UK | Co-investigator | EU-AIMS LEAP |
| Daniel | Brandeis | | Dr | University of Zurich | Zurich, Switzerland | Co-investigator | EU-AIMS LEAP |
| Claudia | Brogna | | Dr | University Campus Bio-Medico | Rome, Italy | Co-investigator | EU-AIMS LEAP |
| Yvette | de Bruijn | | Dr | Radboud University | Nijmegen, Netherlands | Co-investigator | EU-AIMS LEAP |
| Jan K. | Buitelaar | | Dr | Radboud University | Nijmegen, Netherlands | Co-investigator | EU-AIMS LEAP |
| Bhismadev | Chakrabarti | | Dr | University of Reading | Reading, UK | Co-investigator | EU-AIMS LEAP |
| Tony | Charman | | Dr | King's College London | London, UK | Co-investigator | EU-AIMS LEAP |
| Ineke | Cornelissen | | Dr | Radboud University | Nijmegen, Netherlands | Co-investigator | EU-AIMS LEAP |
| Daisy | Crawley | | Dr | King's College London | London, UK | Co-investigator | EU-AIMS LEAP |
| Flavio | Dell'Acqua | | Dr | King's College London | London, UK | Co-investigator | EU-AIMS LEAP |
| Guillaume | Dumas | | Dr | University of Montreal | Montreal, Canada | Co-investigator | EU-AIMS LEAP |
| Sarah | Durston | | Dr | University Medical Center Utrecht | Utrecht, Netherlands | Co-investigator | EU-AIMS LEAP |

Supplemental Online Content: Nonauthor Collaborators
*First name, last name, and suffix (if applicable) are required and will appear in PubMed.

| *First Name and Middle Initial(s) | *Last Name | *Suffix (eg, Jr, III) | Academic Degrees | Institution | Location (city, state/province, country) | Role or Contribution, eg, chair, principal investigator | Group (if more than 1 Group listed in the byline) and/or Subgroup (eg, Steering Committee) |
|---|---|---|---|---|---|---|---|
| Christine | Ecker | | Dr | King's College London | London, UK | Co-investigator | EU-AIMS LEAP |
| Jessica | Faulkner | | Dr | King's College London | London, UK | Co-investigator | EU-AIMS LEAP |
| Vincent | Frouin | | Dr | CEA Neurospin University Paris-Saclay | Paris, France | Co-investigator | EU-AIMS LEAP |
| Pilar | Garcés | | Dr | Complutense University of Madrid | Madrid, Spain | Co-investigator | EU-AIMS LEAP |
| David | Goyard | | Dr | CEA Neurospin University Paris-Saclay | Paris, France | Co-investigator | EU-AIMS LEAP |
| Lindsay | Ham | | Dr | Regulatory Affairs, Pharmaceutical Development, F. Hoffman-La Roche Pharmaceuticalss | Basel, Switzerland | Co-investigator | EU-AIMS LEAP |
| Hannah | Hayward | | Dr | King's College London | London, UK | Co-investigator | EU-AIMS LEAP |
| Joerg | Hipp | | Dr | Roche Pharma Research and Early Development, Roche Innovation Center Basel | Basel, Switzerland | Co-investigator | EU-AIMS LEAP |
| Rosemary | Holt | | Dr | University of Cambridge | Cambridge, UK | Co-investigator | EU-AIMS LEAP |
| Mark H. | Johnson | | Dr | Birkbeck University | London, UK | Co-investigator | EU-AIMS LEAP |
| Emily J.H. | Jones | | Dr | Birkbeck University | London, UK | Co-investigator | EU-AIMS LEAP |
| Prantik | Kundu | | Dr | Icahn School of Medicine at Mount Sinai | New York, NY, USA | Co-investigator | EU-AIMS LEAP |
| Meng-Chuan | Lai | | Dr | University of Cambridge | Cambridge, UK | Co-investigator | EU-AIMS LEAP |
| Xavier | Liogier D'Ardhuy | | Dr | Roche Pharma Research and Early Development, Roche Innovation Center Basel | Basel, Switzerland | Co-investigator | EU-AIMS LEAP |
| Michael V. | Lombardo | | Dr | University of Cambridge | Cambridge, UK | Co-investigator | EU-AIMS LEAP |
| Eva | Loth | | Dr | King's College London | London, UK | Co-investigator | EU-AIMS LEAP |
| David J. | Lythgoe | | Dr | King's College London | London, UK | Co-investigator | EU-AIMS LEAP |
| René | Mandl | | Dr | University Medical Center Utrecht | Utrecht, Netherlands | Co-investigator | EU-AIMS LEAP |

Supplemental Online Content: Nonauthor Collaborators
*First name, last name, and suffix (if applicable) are required and will appear in PubMed.

| ***First Name and Middle Initial(s)** | ***Last Name** | ***Suffix (eg, Jr, III)** | Academic Degrees | Institution | Location (city, state/province, country) | Role or Contribution, eg, chair, principal investigator | Group (if more than 1 Group listed in the byline) and/or Subgroup (eg, Steering Committee) |
|---|---|---|---|---|---|---|---|
| Andre | Marquand | | Dr | Radboud University | Nijmegen, Netherlands | Co-investigator | EU-AIMS LEAP |
| Luke | Mason | | Dr | King's College London | London, UK | Co-investigator | EU-AIMS LEAP |
| Maarten | Mennes | | Dr | Radboud University | Nijmegen, Netherlands | Co-investigator | EU-AIMS LEAP |
| Andreas | Meyer-Lindenberg | | Dr | University of Mannheim | Mannheim, Germany | Co-investigator | EU-AIMS LEAP |
| Carolin | Moessnang | | Dr | University of Mannheim | Mannheim, Germany | Co-investigator | EU-AIMS LEAP |
| Nico | Mueller | | Dr | University of Mannheim | Mannheim, Germany | Co-investigator | EU-AIMS LEAP |
| Declan GM | Murphy | | Dr | King's College London | London, UK | Co-investigator | EU-AIMS LEAP |
| Bethany | Oakley | | Dr | King's College London | London, UK | Co-investigator | EU-AIMS LEAP |
| Laurence | O'Dwyer | | Dr | Radboud University | Nijmegen, Netherlands | Co-investigator | EU-AIMS LEAP |
| Marianne | Oldehinkel | | Dr | Radboud University | Nijmegen, Netherlands | Co-investigator | EU-AIMS LEAP |
| Bob | Oranje | | Dr | University Medical Center Utrecht | Utrecht, Netherlands | Co-investigator | EU-AIMS LEAP |
| Gahan | Pandina | | Dr | Janssen Research & Development | Titusville, NJ, USA | Co-investigator | EU-AIMS LEAP |
| Antonio M. | Persico | | Dr | University of Messina | Messina, Italy | Co-investigator | EU-AIMS LEAP |
| Barbara | Ruggeri | | Dr | King's College London | London, UK | Co-investigator | EU-AIMS LEAP |
| Amber | Ruigrok | | Dr | University of Cambridge | Cambridge, UK | Co-investigator | EU-AIMS LEAP |
| Jessica | Sabet | | Dr | King's College London | London, UK | Co-investigator | EU-AIMS LEAP |
| Roberto | Sacco | | Dr | University Campus Bio-Medico | Rome, Italy | Co-investigator | EU-AIMS LEAP |
| Antonia | San José Cáceres | | Dr | King's College London | London, UK | Co-investigator | EU-AIMS LEAP |
| Emily | Simonoff | | Dr | King's College London | London, UK | Co-investigator | EU-AIMS LEAP |
| Will | Spooren | | Dr | Roche Pharma Research and Early Development, Roche Innovation Center Basel | Basel, Switzerland | Co-investigator | EU-AIMS LEAP |
| Julian | Tillmann | | Dr | King's College London | London, UK | Co-investigator | EU-AIMS LEAP |

Supplemental Online Content: Nonauthor Collaborators
*First name, last name, and suffix (if applicable) are required and will appear in PubMed.

| ***First Name and Middle Initial(s)** | ***Last Name** | ***Suffix (eg, Jr, III)** | Academic Degrees | Institution | Location (city, state/province, country) | Role or Contribution, eg, chair, principal investigator | Group (if more than 1 Group listed in the byline) and/or Subgroup (eg, Steering Committee) |
|---|---|---|---|---|---|---|---|
| Roberto | Toro | | Dr | Institut Pasteur | Paris, France | Co-investigator | EU-AIMS LEAP |
| Heike | Tost | | Dr | University of Mannheim | Mannheim, Germany | Co-investigator | EU-AIMS LEAP |
| Jack | Waldman | | Dr | University of Cambridge | Cambridge, UK | Co-investigator | EU-AIMS LEAP |
| Steve CR | Williams | | Dr | King's College London | London, UK | Co-investigator | EU-AIMS LEAP |
| Caroline | Wooldridge | | Dr | King's College London | London, UK | Co-investigator | EU-AIMS LEAP |
| Marcel | Zwiers | | Dr | Radboud University | Nijmegen, Netherlands | Co-investigator | EU-AIMS LEAP |
