# Supplementary material for: Patterns of Brain Maturation in Autism and Their Molecular Associations
Source: JAMA Psychiatry. 2024 Oct 16;81(12):1253–64. doi: 10.1001/jamapsychiatry.2024.3194 (PMC11581727; doi:10.1001/jamapsychiatry.2024.3194)
Supplement: Supplement 4. — Data Sharing Statement. [file jamapsychiatry-e243194-s004.pdf]

Data Sharing Statement

Pretzsch. Patterns of Brain Maturation in Autism and Their Molecular Associations. *JAMA Psychiatry*. Published October 16, 2024. doi:10.1001/jamapsychiatry.2024.3194

Data
Data available: Yes
Data types: Deidentified participant data, Data dictionary
How to access data: charlotte.pretzsch@kcl.ac.uk
When available: With publication

Supporting Documents
Document types: Other (please specify)
Additional Information: We will share some of the de-identified, preprocessed neuroimaging data.
How to access documents: Data accompanies the manuscript submission.
When available: With publication

Additional Information
Who can access the data: To anyone with access to the manuscript.
Types of analyses: As seen fit by reader
Mechanisms of data availability: In conjunction with the manuscript
